# Effects of Oral L-Arginine on Everyday Persistent Fatigue, Sleep, and Post-Exercise Lactate Recovery in Community-Dwelling Working-Age Adults: A Double-Blind, Placebo-Controlled Randomized Trial

**DOI:** 10.3390/nu18203375

**Published:** 2026-10-14

**Authors:** Eun-Bum Kang, Seong-Bong Hong, Namkyu Yoon, Namju Lee, Byungkwon Kim

**Affiliations:** 1 Department of Sports Health Rehabilitation, Daejeon University, Daejeon 34520, Republic of Korea; kbume23@dju.kr; 2 Department of Sports Data Analysis, Songwon University, Gwangju 61756, Republic of Korea; hsb8604@songwon.ac.kr; 3 Department of Korean Medicine, Daegu Haany University, Daegu 42158, Republic of Korea; yoonnamkyu@dhu.ac.kr; 4 Integrative Medicine Innovation Center, Wonkwang University, Iksan 54538, Republic of Korea; 5 College of Korean Medicine, Wonkwang University, Iksan 54538, Republic of Korea

**Keywords:** L-arginine, persistent fatigue, Fatigue Severity Scale, Pittsburgh Sleep Quality Index, lactate kinetics, exercise recovery, randomized controlled trial

## Abstract

Background/Objectives: The evidence for nutritional interventions against fatigue is limited; we evaluated whether L-arginine reduces fatigue in working-age adults. Methods: In this 8-week randomized, double-blind, placebo-controlled trial, 120 community-dwelling working-age adults aged 30–59 years with persistent fatigue (Fatigue Severity Scale [FSS] total ≥ 27) received oral L-arginine 3 g/day, 6 g/day, or placebo. The primary endpoint was FSS change; secondary endpoints included the Checklist Individual Strength (CIS), Pittsburgh Sleep Quality Index (PSQI), and blood lactate kinetics during a standardized submaximal treadmill run (~70% VO_2_max, 30 min). Data from 119 participants were analyzed, with Holm adjustment for multiplicity. Results: At Week 8, FSS decreased more with 6 g/day than with placebo (−6.41 vs. −2.23; Holm-adjusted *p* = 0.024), whereas 3 g/day did not differ from placebo. The 6 g/day CIS improvement did not survive multiplicity adjustment. PSQI scores improved nominally at both doses (3 g/day, *p* = 0.007; 6 g/day, *p* = 0.045). With 6 g/day, the total lactate area under the curve (Holm-adjusted *p* = 0.041) and 15-min recovery lactate (Holm-adjusted *p* = 0.002) were lower than with the placebo, whereas the resting plasma nitrite did not differ. No serious adverse events occurred. Conclusions: L-arginine supplementation at 6 g/day for 8 weeks reduced fatigue severity relative to placebo and was well-tolerated; however, mean FSS scores remained above the enrollment threshold, indicating a reduction in, rather than resolution of, persistent fatigue.

## 1. Introduction

Persistent fatigue, defined here as fatigue lasting at least one month and encompassing both prolonged (1–6 months) and chronic (≥6 months) fatigue [1], is a prevalent condition that significantly impairs daily functioning, and its burden is exacerbated in societies characterized by chronic insufficient recovery. In South Korea, annual hours worked per employed person were 1901 in 2022, 149 h more than the average of 1752 h across Organization for Economic Co-operation and Development (OECD) countries, placing the population in an environment with high structural risks for fatigue [2]. Consistent with these conditions, 21.1% of adults in community primary care report prolonged fatigue, and 8.4% report chronic fatigue, with psychological stress identified as the primary contributor [1]. Furthermore, in South Korea, the prevalence of chronic fatigue syndrome was reported to have increased approximately 1.94-fold between 2010 and 2020 [3].

At the same time, many adults engage in routine leisure-time physical activities, such as jogging or running, to restore vitality and improve cardiometabolic health, with approximately 60% of South Korean adults participating in organized sports at least once a week [4]. However, acute fatigue and delayed recovery following unaccustomed exercise loads can hinder consistent participation and daily functioning. Thus, there is a growing demand for scalable, non-pharmacological, evidence-based, and mechanism-informed nutritional strategies that can support both chronic daily fatigue and post-exercise recovery, applicable outside of specialized clinical settings.

Fatigue associated with chronic sleep restriction and psychosocial stress is linked to endothelial dysfunction and reduced nitric oxide (NO) bioavailability, which are mediated by sympathetic overactivity, elevated oxidative stress, and increased levels of asymmetric dimethylarginine (ADMA) [5], an endogenous inhibitor of endothelial nitric oxide synthase (eNOS) [6,7]. Some forms of fatigue may share the modifiable pathway of reduced NO signaling, offering a rational intervention target. L-arginine, as an obligate substrate for eNOS, may promote endothelium-dependent vasodilation under appropriate conditions, thereby redistributing blood flow to active muscles and assisting in oxygen delivery and metabolite clearance during and after exercise [8,9]. However, circulating plasma NO is a systemic snapshot with a short half-life and high variability, failing to sensitively reflect local, activity-dependent endothelial NO signaling. Moreover, the arginine paradox, whereby exogenous L-arginine enhances eNOS-dependent NO production even when intracellular substrate saturation is near-maximal and plasma arginine levels are within the normal range, suggests that functional benefits may occur without marked changes in systemic NO concentrations [10,11,12].

Clinical findings for oral L-arginine remain heterogeneous, characterized by differences in study populations, dosages, durations, and baseline NO levels, and rely heavily on vascular surrogates, biomarkers, or specific disease cohorts. While short-term administration in patients with cardiometabolic disease has shown improvements in flow-mediated dilation (FMD) in some studies, others have reported no effect, and safety concerns have been raised regarding its use following acute myocardial infarction [13,14,15]. Such inconsistencies suggest the need to evaluate functional outcomes, specifically symptoms and objective recovery, in non-acute and representative populations rather than relying solely on circulating NO biomarkers. In this study, we treated plasma nitrite as an exploratory biomarker with inherent limitations in sensitivity, focusing instead on post-exercise recovery kinetics as a functional test of the perfusion hypothesis. Specific mechanistic predictions are stated at the end of this section; a failure to observe the recovery-phase improvement would weigh against a perfusion-mediated interpretation.

The evidence regarding exercise-related outcomes is similarly conflicting. While small, well-controlled studies suggest that acute L-arginine can reduce oxygen consumption during submaximal exercise and increase high-intensity endurance [16], other reports indicate no effect on performance or NO biomarkers, and meta-analyses document significant heterogeneity in aerobic capacity improvements [17,18]. Critically, there remains a gap regarding the target population. Previous research on L-arginine for fatigue has predominantly focused on clinical cohorts, such as patients with long COVID or elderly post-COVID patients [19], or on trained athletes. Conversely, randomized controlled trials evaluating single-ingredient L-arginine in community-dwelling adults without specific underlying diseases, who experience subclinical daily fatigue (FSS total score ≥ 27, i.e., item mean ≥ 3; below the threshold of FSS total ≥ 36 [item mean ≥ 4] commonly used to indicate clinically significant fatigue [20]), remain limited [21]. This study was designed to enhance generalizability by using this cutoff to compose a sample representative of community-dwelling adults engaging in leisure activities.

Therefore, the primary objective of this study was to verify whether 8 weeks of L-arginine supplementation reduces fatigue (FSS) compared to a placebo in community-dwelling adults with persistent fatigue (FSS total score ≥ 27). The secondary objective was to examine, as a separate mechanistic endpoint, whether L-arginine alters blood lactate recovery after a standardized submaximal exercise bout. We conducted an 8-week randomized, double-blind, placebo-controlled trial comparing L-arginine (3 g/day or 6 g/day) with a placebo. We assessed validated patient-reported outcomes, including the Fatigue Severity Scale (FSS) [20], the Checklist Individual Strength (CIS) [22], and the Pittsburgh Sleep Quality Index (PSQI) [23], and evaluated blood-lactate pharmacokinetics (area under the concentration–time curve [AUC], maximum observed concentration [Cmax], time of maximum concentration [Tmax], and pre-/post-peak AUC) following a standardized submaximal treadmill run (~70% VO_2_max, 30 min) [24,25]. Based on the mechanism that sufficient L-arginine supplementation increases eNOS substrate availability to promote perfusion and metabolite clearance during the recovery phase, we established the following hypotheses: 1. L-arginine reduces FSS fatigue at 8 weeks to a greater extent than placebo in a dose-dependent manner (6 g/day > 3 g/day > placebo). 2. Any reduction in post-exercise lactate would be predominantly confined to the recovery phase, reflected in a lower 15-min recovery lactate and a lower immediate post-exercise lactate, without changes in pre-peak (production-phase) AUC, time-to-peak (Tmax), or glucose. The pre-/post-peak AUC decomposition itself was specified as an exploratory analysis, with its phase-specific metrics (post-peak AUC and Cmax) treated as supportive rather than confirmatory.

## 2. Materials and Methods

### 2.1. Participants and Eligibility

**Figure 1:**
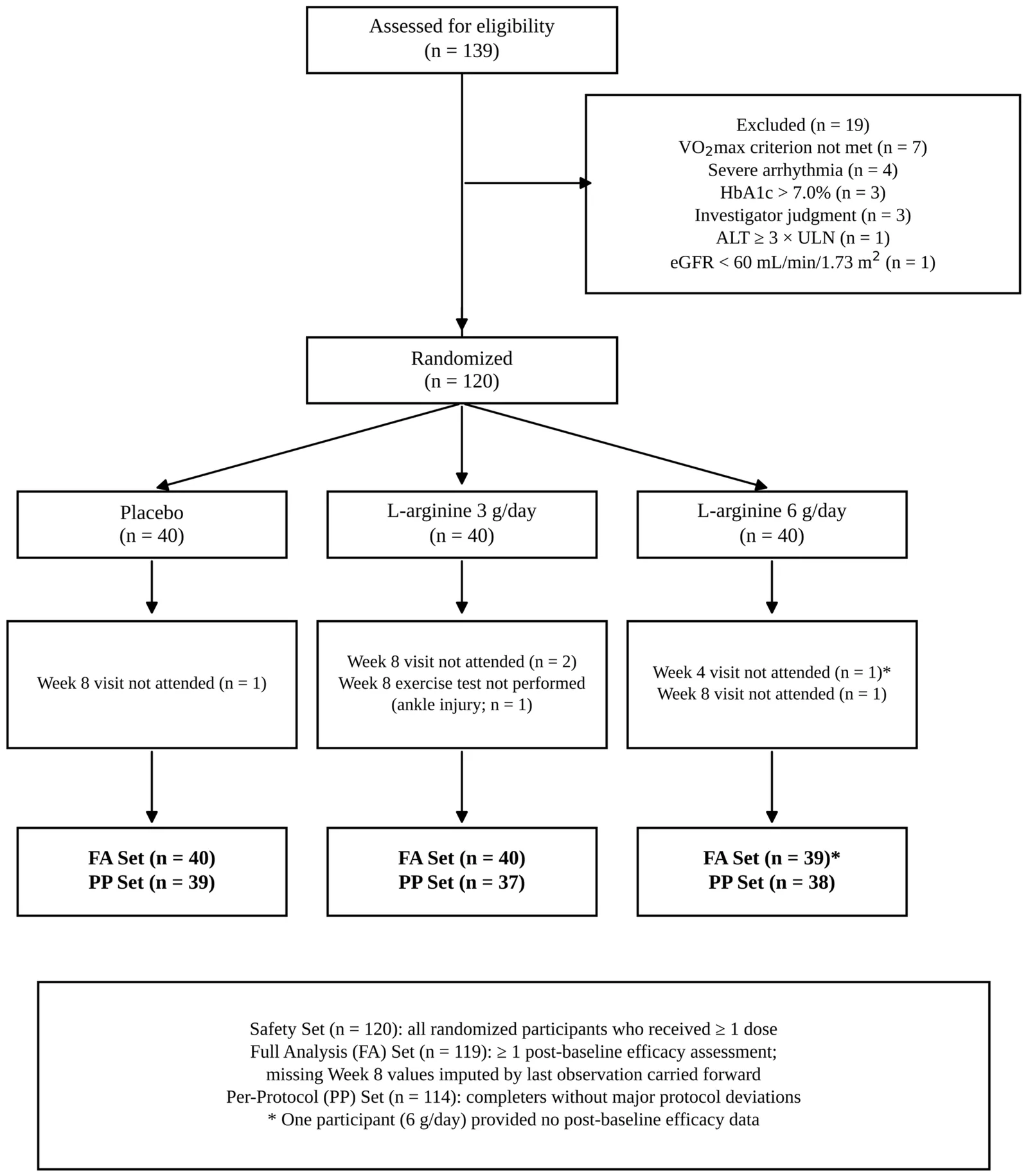
Consolidated Standards of Reporting Trials (CONSORT) flow of participants and analysis sets. A total of 139 adults were assessed for eligibility; 120 were randomized 1:1:1 to placebo, L-arginine 3 g/day, or L-arginine 6 g/day (40/40/40). All randomized participants formed the Safety set (*n* = 120). One participant (6 g/day) lacked any post-baseline efficacy data, yielding the Full Analysis (FA) set (*n* = 119). The Per-Protocol (PP) set (*n* = 114) excluded four for nonattendance at Week 8 and one unable to perform the exercise test. Final PP numbers: placebo (*n* = 39), L-arginine 3 g/day (*n* = 37), L-arginine 6 g/day (*n* = 38).

This randomized, double-blind, placebo-controlled study was approved by the Institutional Review Board (IRB) of Daejeon University (Approval No. 1040647-202404-HR-005-03; approved 15 May 2024). This trial was registered with the Clinical Research Information Service (CRIS; https://cris.nih.go.kr, accessed on 14 July 2026), Republic of Korea (Registration No. KCT0012357), on 30 July 2026; registration was completed retrospectively, after completion of the study, whereas the protocol and its primary endpoint (FSS total score) had been approved by the IRB before enrolment began. The trial was conducted at Cheongchun Hospital (Daejeon, Republic of Korea) in compliance with the Declaration of Helsinki and the Korean Good Clinical Practice (KGCP) guidelines. All participants provided written informed consent prior to study enrollment. Recruitment occurred between 15 July 2024, and 13 December 2024. A total of 120 participants were enrolled (40 per group), accounting for a 20% anticipated attrition rate (Figure 1). All screening, randomization, intervention oversight, and physiological testing were performed at the study site.

Participants were community-dwelling working-age adults, i.e., recruited from the general community and living independently rather than in clinical or institutional settings. Inclusion criteria were adults aged 30–59 years with a Fatigue Severity Scale (FSS) total score ≥ 27 and a VO_2_max between the 25th and 85th percentiles of Korean normative data (i.e., individuals in the top 15% or bottom 25% were excluded) [26]. Exclusion criteria included recent use of products influencing fatigue or exercise, substance abuse, hepatic dysfunction (aspartate or alanine aminotransferase [AST/ALT] ≥ 3 × the upper limit of normal [ULN] or bilirubin ≥ 2 × ULN), estimated glomerular filtration rate (eGFR) < 60 mL/min/1.73 m^2^, uncontrolled diabetes (glycated hemoglobin [HbA1c] > 7%), blood pressure abnormalities (hypotension: systolic/diastolic blood pressure [SBP/DBP] < 90/60 mmHg; hypertension: SBP/DBP > 140/90 mmHg), major chronic diseases (e.g., cancer, tuberculosis, multiple sclerosis), uncontrolled psychiatric disorders, extreme body mass index (BMI) (≤18.5 or ≥35 kg/m^2^), night-shift work, pregnancy/lactation, or excessive alcohol/smoking.

Participants were discontinued from the study in cases of protocol violations, participant request, occurrence of adverse events (including serious adverse events [SAEs]), non-adherence (<80% average intake), or the use of prohibited products or therapies without prior approval. Reasons for discontinuation were recorded in the case report form (CRF), and participant flow and baseline characteristics are presented in Section 3 (see Figure 1).

### 2.2. Experimental Design

This was an 8-week, randomized, double-blind, placebo-controlled, parallel-group clinical study with three arms allocated in equal proportions (L-arginine 3 g/day, L-arginine 6 g/day, or placebo). Randomization was performed using a computer-generated sequence with a 1:1:1 allocation ratio and a block size of 6 to ensure group balance. Screening and randomization numbers were issued sequentially according to the study protocol.

#### 2.2.1. Allocation Concealment and Masking

The randomization code was maintained by an independent custodian and remained sealed until study completion. Subject-specific sealed envelopes were prepared for potential emergencies to allow for individual unblinding if necessary; however, no emergency unblinding was required. To ensure blinding for participants and site staff, active and placebo products were provided in identical labels and packaging. Participants, investigators, and outcome assessors remained blinded to group assignment throughout the study.

#### 2.2.2. Visit Schedule and Assessment Windows

Participants attended 4 protocol visits: Screening (Visit 1), Baseline (Visit 2), Week 4 (Visit 3), and Week 8 (Visit 4). For on-treatment visits, a window of ±5 days was permitted. The schedule of assessments is summarized in Supplementary Table S1 and included the following: questionnaires (Visits 2, 3, and 4); the Korean version of the Pittsburgh Sleep Quality Index (PSQI-K; Visits 2 and 4); clinical laboratories (Visits 1 and 4); and vital signs and pregnancy tests (all visits). At Visits 2 and 4, participants underwent a standardized treadmill challenge, with the rating of perceived exertion (RPE) and the fatigue visual analogue scale (VAS) collected around the exercise bout and biospecimen sampling (lactate, glucose, lactate dehydrogenase [LDH], and cortisol) performed pre-exercise, immediately post-exercise, and at 15 and 30 min of recovery. Plasma nitrite (an NO oxidation product) was also measured at Visits 2 and 4.

### 2.3. Interventions, Control, and Adherence

#### 2.3.1. Investigational Products (IPs) and Dosing

Three visually matched sachet products were used: 2 liquid L-arginine products and a matched placebo. Trial Food I contained 1.50 g of L-arginine per 20 g sachet, while Trial Food II contained 3.00 g of L-arginine per 20 g sachet; both contained purified water, tartaric acid (1.30 g and 1.80 g per sachet, respectively), sucralose, lemon–orange flavor, and xanthan gum. Trial Food III (placebo) consisted of a 20 g sachet containing 0 g of L-arginine with the same excipients, except that it contained 0.30 g of tartaric acid and 0.10 g of dextrin. All IPs were manufactured and provided by Daesang Wellife Co., Ltd. (Seoul, Republic of Korea) Products were stored in a cool, dry place, protected from direct light, with a validated 24-month shelf life. Participants consumed one sachet twice daily for 8 weeks, resulting in a total daily intake of 3 g in the lower-dose arm (Trial Food I), 6 g in the higher-dose arm (Trial Food II), or the equivalent placebo (Trial Food III).

#### 2.3.2. Dispensation, Returns, and Accountability

Study products were dispensed at Baseline (Visit 2) and Week 4 (Visit 3), and collected and reconciled at Week 4 (Visit 3) and Week 8 (Visit 4). The IP manager recorded issuance (date, quantity, and participant ID) in inventory logs and performed sachet counts upon return. A monitor periodically cross-checked these logs against on-site inventory. The operational timeline is summarized in Supplementary Table S1.

#### 2.3.3. Adherence Definition and Thresholds

Compliance was computed as a percentage: Compliance (%) = (number of sachets taken ÷ number of sachets planned) × 100. Per protocol, an average intake of <80% was a prespecified criterion for study discontinuation and exclusion from the per-protocol (PP) set for efficacy analyses; in practice, no participant had a mean adherence below 80% (minimum 81.2%), and no participant was discontinued for non-adherence. Compliance did not differ between either active group and placebo, although Week 4 compliance was slightly higher at 3 g/day than at 6 g/day (93.2% vs. 89.4%, *p* = 0.006), with both means well above 80% (Table 1).

#### 2.3.4. Concomitant Therapy Control

Participants’ ongoing treatments or supplements at baseline were documented, and they were counseled to avoid initiating or modifying any medication or supplement during the trial. The use of concomitant medications or therapies was discouraged unless medically necessary and unlikely to interfere with the investigational product (IP). Any such use required investigator approval and formal documentation. To minimize the confounding of fatigue or exercise outcomes, the protocol explicitly prohibited the use of specific functional ingredients (e.g., ginseng/red ginseng, Rhodiola, Schisandra, fermented oyster extract, and turmeric extract).

### 2.4. Data Collection and Processing

#### 2.4.1. Endpoints and Instruments

The primary endpoint was the total score of the Fatigue Severity Scale (FSS), a nine-item instrument scored from 1 to 7 per item, where higher scores indicate greater fatigue [20]. The Korean-validated version of the FSS was utilized [27]. Additional patient-reported fatigue measures included the Checklist Individual Strength (CIS; a 20-item, seven-point scale comprising four subscales: subjective fatigue, concentration, motivation, and physical activity) [22,28], the Multidimensional Fatigue Scale (MFS; a seven-point scale comprising three subscales: global fatigue, daily-life dysfunction, and situational fatigue, alongside a total score) [29], and a fatigue VAS. Sleep quality was assessed with the validated PSQI-K [23,30], which provides a global score and seven component scores [31]. Objective exercise-recovery physiology was evaluated during a standardized treadmill challenge, involving serial blood sampling for lactate, glucose, LDH, and cortisol. Nitric oxide status was assessed as plasma nitrite (an NO oxidation product) at rest, sampled at Weeks 0 and 8 independently of the exercise bout, and is reported in µmol/L. Safety assessments included hematology, chemistry, lipids, urinalysis, vital signs, pregnancy testing (where applicable), and adverse events. The full registry of endpoints is provided in Supplementary Table S1.

#### 2.4.2. Visit Schedule and Timing Windows

Assessments were conducted at Screening (Visit 1), Baseline/Week 0 (Visit 2), Week 4 (Visit 3), and Week 8 (Visit 4), with a window of ±5 days allowed for on-treatment visits. The standardized treadmill exercise challenge, including serial blood sampling, was performed at Visits 2 and 4. A consolidated schedule of these assessments is provided in Supplementary Table S1.

#### 2.4.3. Exercise-Challenge Sampling Frame

During the treadmill exercise protocol at Visits 2 and 4, blood samples were collected pre-exercise, immediately post-exercise, and at 15 and 30 min of recovery. The measured analytes included lactate, glucose, LDH, and cortisol. These time-point labels are used consistently throughout the results tables. This sampling frame was adopted from a previous randomized controlled trial that applied an equivalent treadmill protocol (30 min at 70% VO_2_max) to adults aged 30–60 years with FSS ≥ 27 and that also served as the reference study for our sample size calculation [32]. The sampling frame therefore characterizes blood lactate during the first 30 min of recovery; it did not include the 3–8-min window in which blood lactate typically peaks after exercise [25]; by 15 min, lactate had declined to 1.6–1.8 mmol/L, approaching resting values by 30 min.

#### 2.4.4. Exercise-Testing Protocols (VO_2_max Determination and Fatigue Induction)

Maximal oxygen uptake (VO_2_max) was determined at screening (Visit 1) using an incremental maximal treadmill exercise test based on the modified Bruce protocol. Respiratory gas analysis (breath-by-breath) and 12-lead electrocardiographic monitoring were conducted (CASE Exercise Testing System, GE HealthCare, Milwaukee, WI, USA) using a motorized treadmill (9100T, STEX Inc., Seoul, Republic of Korea). The test was terminated and VO_2_max recorded upon the fulfillment of at least three of the following four criteria: a plateau in VO_2_ despite an increasing workload, a respiratory exchange ratio ≥ 1.15, attainment of the maximal breathing rate (35 breaths/min), or an RPE of 19–20 on the Borg 6–20 scale [33]. These strict, sex- and age-adjusted termination criteria adhere to established recommendations [34].

At Baseline (Visit 2) and Week 8 (Visit 4), acute exercise-induced fatigue was elicited using a standardized submaximal treadmill running protocol adapted from Cabral-Santos et al. [35]. Following an incremental lead-in, treadmill speed and grade were progressively increased until participants reached 70% of their individual VO_2_max, an intensity corresponding to a “somewhat hard” rating of 12–14 on the Borg 6–20 scale [33]. This workload was then maintained for a total of 30 min of running (NR20X, DRAX, Tokyo, Japan). RPE was recorded every 5 min (seven time points) throughout the bout. Participants unable to sustain 70% of their VO_2_max for the full 30 min were classified as having failed the standardized physical-stress protocol and were managed according to prespecified procedures. The blood-sampling frame for this exercise bout is described above. Consistent with the previous trial that used the same assessment structure [32], VO_2_max was measured only at screening. A post-intervention maximal graded exercise test was considered during protocol development but was not included, because performing it within the visit window, only a few days before the Week 8 recovery test, could leave residual fatigue, muscle damage, or acute adaptation that would confound the recovery-phase lactate kinetics. The Week 8 challenge was therefore performed at the same workload as at baseline (70% of baseline VO_2_max) to compare recovery kinetics under identical conditions.

#### 2.4.5. Blood Lactate Measurement

Blood lactate was measured using a dedicated enzymatic-amperometric analyzer equipped with an immobilized lactate-oxidase chip biosensor (Biosen C-Line, EKF Diagnostics, Barleben, Germany). A 20 µL whole-blood sample was dispensed into a hemolyzing solution for analysis. This solution lyses erythrocytes and halts glycolysis, thereby precluding in vitro lactate drift. Lactate concentrations are reported in mmol/L (measurement range: 0.5–40 mmol/L; coefficient of variation: ≤3%). The Biosen enzymatic-amperometric method is a validated technique for blood-lactate assessment. Other exercise-associated analytes (glucose, LDH, and cortisol) were processed separately: venous blood was centrifuged, and the resulting plasma/serum was aliquoted and stored at −80 °C prior to analysis at an external specialized clinical laboratory. Resting plasma nitrite, a systemic mechanistic biomarker, was sampled at baseline and Week 8, independent of the exercise bout, and processed using the same standardized procedure.

#### 2.4.6. Derived Variables and Scoring

For all questionnaires, total and subscale scores were calculated according to the respective instrument manuals (FSS, CIS, MFS, VAS, and PSQI-K). Regarding exercise biomarkers, pharmacokinetic-style summaries were derived, including the area under the concentration–time curve (AUC) for overall exposure, the maximum observed concentration (Cmax) for peak, and the time of maximum concentration (Tmax) for time-to-peak. AUC values were calculated using the linear trapezoidal rule in APL PKMP V1.03.52 (PL Analytics, Eden Prairie, MN, USA). In addition to total AUC, pre-peak and post-peak AUC for lactate were computed by splitting each profile at the subject-specific Tmax, allowing for the localization of changes during the ascending versus recovery phases. Cmax and Tmax were determined by direct observation of each participant’s time-concentration profile.

#### 2.4.7. Data Capture and Quality Control

Source data were transcribed into electronic case report forms (eCRFs) within an electronic data capture (EDC) system. On-site monitoring was conducted to verify source-to-eCRF accuracy and protocol adherence. Additionally, remote EDC monitoring, complemented by query management, provided ongoing data validation. Monitoring activities were summarized in the Clinical Study Report (CSR) monitoring log. Relevant protocol amendments affecting data handling (e.g., clarifications to blood-draw procedures and AUC software specifications) are documented in the CSR; the methods described herein reflect the final, implemented procedures.

### 2.5. Statistical Analysis

#### 2.5.1. Sample Size Calculation

The sample size was calculated based on the between-group difference in the Fatigue Severity Scale (FSS) change reported in a previous randomized trial of functional foods for fatigue, which indicated a mean difference of 5 points with a standard deviation of 7 [32]. Assuming a two-sided α of 0.05 and a power of 80% (1 − *β* = 0.80) for comparing each active arm with the placebo in a 1:1:1 parallel-group design, 31 participants per group were required. Accounting for a 20% attrition rate and using a randomization block size of 6, the target enrollment was set at 40 participants per group, resulting in a total planned sample size of 120 participants across the three study arms.

#### 2.5.2. Analysis Populations and General Conventions

Three analysis populations were defined: a Safety Set (all randomized participants who ingested ≥1 dose), a Full Analysis (FA) Set (all randomized participants with at least one post-baseline efficacy assessment), and a Per-Protocol (PP) Set (participants who completed the study without major protocol violations). In the study protocol and clinical study report, the PP Set was designated as the primary efficacy population and the FA Set, in which missing post-baseline values were imputed by last observation carried forward (LOCF), as the supportive population. For this report, the FA Set is presented as the primary analysis population to align with intention-to-treat principles (ICH E9, CONSORT); this was a post hoc change, and the PP Set results are presented as a sensitivity analysis. Conclusions for the primary endpoint were identical in both sets. Safety outcomes were analyzed in the Safety Set.

All analyses were performed using IBM SPSS Advanced Statistics (version 29). Unless otherwise stated, statistical tests were two-sided with an α of 0.05. *p* values are reported to three decimal places, while means, standard deviations, and percentages are reported to two decimal places (extended to three if necessary for clarity). The normality of continuous variables was assessed prior to parametric testing. Equal 1:1:1 randomization was adopted to ensure balanced statistical precision across all pairwise contrasts, including the between-dose comparison (3 g/day vs. 6 g/day).

Because the 2 active arms were compared with a shared placebo across several endpoints, between-group contrasts were adjusted for multiplicity within each endpoint family using the Holm sequentially rejective procedure [36]. Four endpoint families were defined, each including the contrasts of both active arms versus placebo: the fatigue family (FSS total score; two contrasts), the CIS family (CIS total score; two contrasts), the lactate time-point family (15- and 30-min recovery values; four contrasts), and the lactate pharmacokinetic family (total AUC, post-peak AUC, and Cmax; six contrasts). The immediate post-exercise value was reported descriptively alongside Cmax, and pre-peak AUC and time-to-peak were reported as descriptive production-phase parameters. PSQI outcomes were treated as exploratory secondary endpoints and are reported with nominal *p* values. Because this adjustment was applied post hoc, both nominal and Holm-adjusted *p* values are reported. Confirmatory statements are based on the adjusted values, while endpoints that did not survive adjustment are interpreted as supportive or exploratory (hypothesis-generating). Results in the FA and PP Sets were concordant for the primary endpoint and the lactate endpoints; discrepancies were confined to secondary or exploratory endpoints at 3 g/day (the CIS total score and concentration subscale reached significance in the PP Set only, whereas RPE at 20 min reached significance in the FA Set only). Detailed per-protocol results are provided in Supplementary Tables S7 and S8.

#### 2.5.3. Descriptive Summaries and Baseline Comparisons

**Figure 2:**
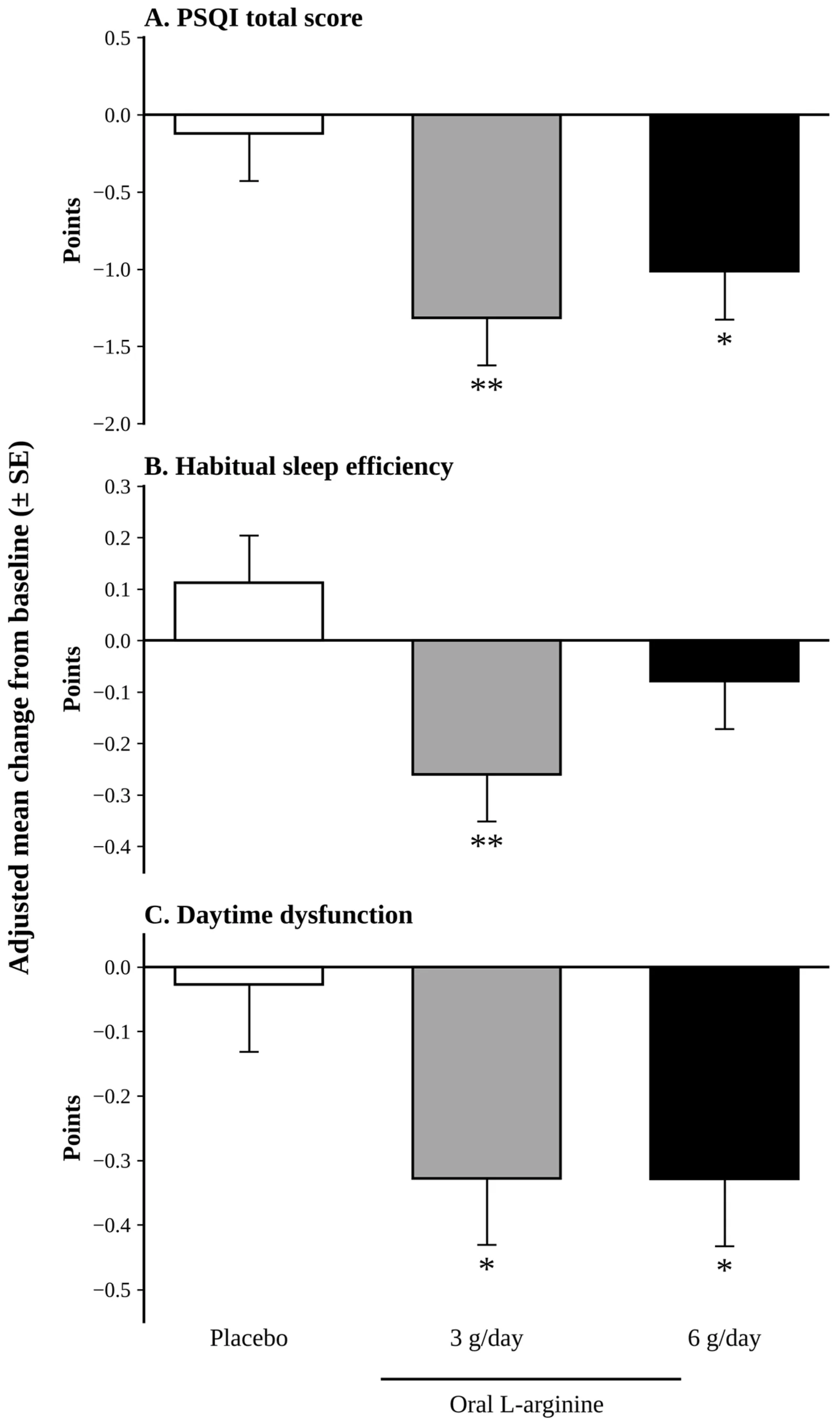
Sleep quality (PSQI) at Week 8 (full analysis set). Baseline-adjusted mean change from baseline (±SE) in (**A**) PSQI total score, (**B**) habitual sleep efficiency, and (**C**) daytime dysfunction, analyzed by analysis of covariance (ANCOVA) with the corresponding baseline value entered as a covariate. Lower PSQI scores indicate better sleep; thus, a negative change reflects improvement. Because daytime dysfunction (**C**) showed a baseline imbalance (Table 2), ANCOVA was applied to this domain as permitted by the statistical analysis plan; the same model was applied post hoc to the total score (**A**) and habitual sleep efficiency (**B**) for consistency. All panels are component or total scores in points. Between-group effects are reported as adjusted mean differences versus placebo. Asterisks denote nominal (unadjusted) significance versus placebo (* *p* < 0.05; ** *p* < 0.01).

**Figure 3:**
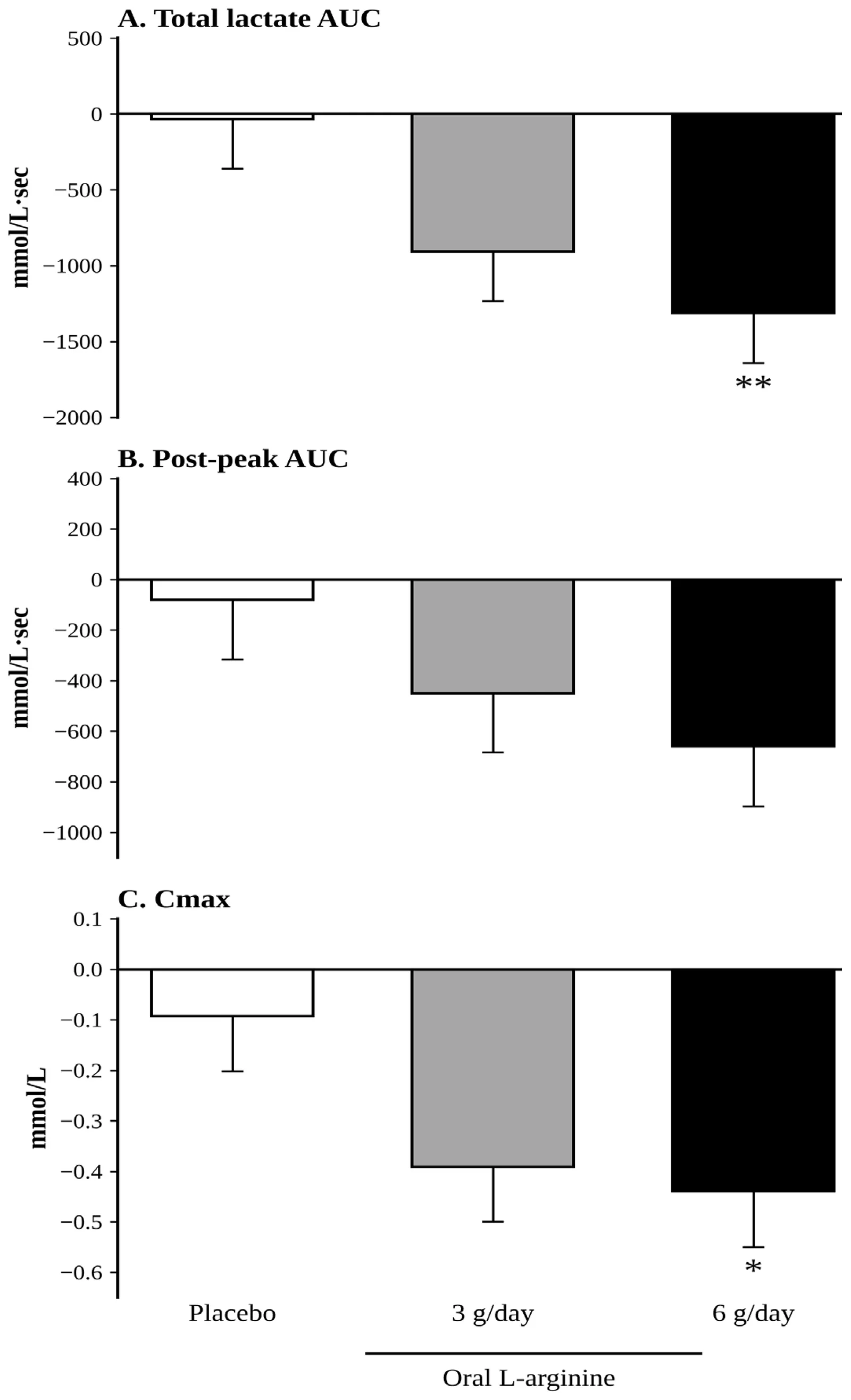
Lactate pharmacokinetic indices during the treadmill challenge at Week 8 (full analysis set). Baseline-adjusted mean change from baseline (±SE), estimated by ANCOVA with the baseline value as a covariate, for (**A**) total lactate AUC (mmol/L·s), (**B**) post-peak AUC (mmol/L·s), and (**C**) Cmax (mmol/L). Lower (more negative) values indicate reduced lactate exposure (AUC) or peak (Cmax). Asterisks denote nominal significance versus placebo (* *p* < 0.05; ** *p* < 0.01); after Holm adjustment within the pharmacokinetic family, only total AUC at 6 g/day remained significant (adjusted *p* = 0.041). The 3 g/day group showed no between-group difference.

Continuous variables are presented as N, mean ± standard deviation (SD), and categorical variables as *n* (%). For baseline characteristics, within-group changes used paired *t*-tests (or Wilcoxon signed-rank if non-normal), between-group comparisons used independent *t*-tests (or Mann–Whitney U if non-normal), and categorical data used χ^2^ (or Fisher’s exact when appropriate). No stratified or subgroup analyses were required, because baseline characteristics were balanced across groups. The statistical analysis plan permitted baseline-adjusted analysis of covariance (ANCOVA), with the baseline value entered as a covariate, for variables showing a between-group difference at baseline; on this basis, ANCOVA was applied to the PSQI daytime dysfunction component, which was imbalanced at baseline (Table 2). For consistency, the same baseline-adjusted ANCOVA was additionally applied post hoc to the PSQI total score and habitual sleep efficiency (Figure 2) and to the lactate time-point and pharmacokinetic endpoints (Figure 3); these results are reported as adjusted mean differences with 95% confidence intervals (CIs). Unadjusted change-from-baseline comparisons (*t*-test or Mann–Whitney U test) are shown in Table 3, Table 4, Table 5 and Table 6 for transparency.

#### 2.5.4. Primary Endpoint and Other Patient-Reported Outcomes

For efficacy endpoints (the primary FSS endpoint and secondary CIS, MFS, VAS, and PSQI endpoints), the analysis proceeded as follows: (i) normality assessment; (ii) presentation of descriptive summary statistics; (iii) within-group change from baseline assessed by paired *t*-tests or Wilcoxon signed-rank tests; (iv) between-group comparisons versus placebo at each scheduled visit (Week 4 and Week 8) using independent *t*-tests or Mann–Whitney U tests; and (v) comparison of change-from-baseline at Week 8 between each active arm and placebo using the aforementioned tests.

#### 2.5.5. Exercise-Recovery Biomarkers and Pharmacokinetic-Style Summaries

Serial analytes collected during the treadmill challenge (lactate, glucose, LDH, and cortisol) were summarized at pre-exercise, immediately post-exercise, and at 15 and 30 min of recovery. To quantify exposure and peak dynamics, the area under the concentration–time curve (AUC), maximum observed concentration (Cmax), and time of maximum concentration (Tmax) were calculated. AUC values were determined using the linear trapezoidal rule implemented in APL PKMP (version 1.03.52; APL Analytics, Eden Prairie, MN, USA). Cmax and Tmax were derived via direct observation of each subject’s time–concentration curve. Because sampling was limited to four time points, Cmax and Tmax denote the highest concentration observed among the sampled time points and its timing, respectively, rather than the true physiological peak.

#### 2.5.6. Handling of Missing Data and Analysis-Set Preferences

The FA Set (presented post hoc as the primary analysis set) was analyzed with missing post-baseline values imputed by LOCF, consistent with the clinical study report; this applied to the four participants who did not attend the Week 8 visit and, for the exercise-challenge endpoints, to the participant unable to perform the Week 8 exercise test. The PP Set, which required no imputation, was used for sensitivity analysis. Baseline characteristics were summarized for both sets, with the PP Set provided in Supplementary Tables S6–S8.

#### 2.5.7. Safety Analyses

Adverse events were tabulated by system organ class and preferred term. χ^2^ tests (or Fisher’s exact tests when appropriate) were conducted to compare the frequencies and proportions of adverse events between groups. Clinical laboratory results (hematology, chemistry, lipids, and urinalysis), vital signs, and pregnancy test results were summarized descriptively, with pre- versus post-treatment comparisons performed where applicable.

## 3. Results

### 3.1. Participant Characteristics and Analysis Populations

A total of 139 individuals were screened, and 120 were randomized in equal strata to placebo, 3 g/day L-arginine, or 6 g/day L-arginine (40 participants per group). The Safety Set comprised all randomized participants who ingested at least one dose (*n* = 120). The Full Analysis (FA) Set, the primary analysis population, comprised all randomized participants with at least one post-baseline efficacy assessment (*n* = 119; one participant in the 6 g/day group provided no post-baseline data). The Per-Protocol (PP) Set, used for sensitivity analysis, comprised study completers without major protocol violations (*n* = 114), following the exclusion of four participants who did not attend the Week 8 (Visit 4) assessment and one participant unable to perform the exercise test (Figure 1).

The results from the PP Set were concordant with the primary analysis for the primary endpoint and the lactate endpoints. Discrepancies were confined to secondary or exploratory endpoints at 3 g/day: the Checklist Individual Strength (CIS) total score and concentration subscale reached significance in the PP Set but not in the FA Set, whereas RPE at 20 min reached significance in the FA Set only. Per-protocol results are provided in Supplementary Tables S7 and S8.

No statistically significant differences in baseline characteristics were observed across the three groups, or between the two active arms (3 g/day vs. 6 g/day), among the baseline characteristics shown in Table 2, with the single exception of the PSQI daytime dysfunction component, which was higher in both active arms than with placebo (*p* = 0.007 and *p* = 0.023) and was therefore analyzed by baseline-adjusted ANCOVA (Table 2). Baseline VO_2_max was also balanced across groups, including between the two active arms (3 g/day vs. 6 g/day, *p* = 0.367; Table 2). These values fall within the 25th–85th percentiles of Korean age- and sex-specific normative data [26], the range required for enrollment, indicating that participants had normal baseline cardiorespiratory fitness. Medical history and medication use/changes were similarly balanced (Supplementary Table S2). Lifestyle factors, including alcohol, smoking, caffeine, and sugar/aspartame intake, did not differ between groups (Supplementary Tables S3 and Table S4). Furthermore, physical activity (Global Physical Activity Questionnaire [GPAQ]; Table 2) and dietary intake showed no material between-group differences (Supplementary Tables S4 and S5). Study product adherence was high in all groups (mean adherence 91–93% at Week 8) and did not differ between either active group and placebo; at Week 4, adherence was slightly higher at 3 g/day than at 6 g/day (93.2% vs. 89.4%, *p* = 0.006), with both means well above 80% (Table 1). Baseline values for all efficacy variables are provided in Supplementary Table S6.

Because the trial involved two active-versus-placebo comparisons across several endpoints, between-group contrasts were adjusted for multiplicity within each endpoint family using a post hoc Holm procedure. Both nominal and Holm-adjusted *p* values are reported, and the latter serve as the basis for all confirmatory statements.

### 3.2. Fatigue Outcomes: Primary (FSS) and Supportive (CIS)

In the Full Analysis (FA) Set, 6 g/day of L-arginine significantly reduced total Fatigue Severity Scale (FSS) scores compared to placebo at both Week 4 and Week 8. At Week 4, the mean change from baseline was −3.87 ± 4.62 in the 6 g/day group versus −1.18 ± 3.68 in the placebo group (*p* = 0.006). At Week 8, the mean change was −6.41 ± 8.02 versus −2.23 ± 6.02, respectively (*p* = 0.012). This primary effect remained significant after Holm adjustment (adjusted *p* = 0.024 at Week 8), indicating that 6 g/day reduced fatigue relative to placebo. The 3 g/day group did not differ significantly from placebo at either time point (*p* = 0.980 at Week 4; *p* = 0.935 at Week 8). The FSS total score and its nine individual items are summarized in Table 3.

Regarding the supportive Checklist Individual Strength (CIS) scale, 6 g/day of L-arginine improved the CIS total score compared to placebo at Week 8 (nominal *p* = 0.029), with significant within-group reductions from baseline observed in the 6 g/day group. However, this effect did not survive Holm adjustment within the CIS family and is therefore interpreted as supportive rather than confirmatory (Table 4).

### 3.3. Sleep Outcomes: Pittsburgh Sleep Quality Index (PSQI)

The PSQI was assessed at Week 0 and Week 8, with lower scores indicating better sleep quality. Observed change-from-baseline values are presented in Table 4. Baseline-adjusted results for the total score, habitual sleep efficiency, and daytime dysfunction, analyzed by analysis of covariance (ANCOVA) with the corresponding baseline value entered as a covariate, are shown in Figure 2. Between-group effects are reported as adjusted mean differences versus placebo with 95% confidence intervals (CIs).

At Week 8, both L-arginine doses significantly improved the PSQI total score relative to placebo, at 3 g/day (adjusted mean difference −1.19 points, 95% CI −2.06 to −0.33; *p* = 0.007) and at 6 g/day (−0.89 points, 95% CI −1.77 to −0.02; *p* = 0.045), with overlapping confidence intervals indicating no clear separation between the two doses. For habitual sleep efficiency, 3 g/day improved versus placebo (adjusted mean difference −0.37, 95% CI −0.63 to −0.11; *p* = 0.005), whereas 6 g/day did not (−0.19, 95% CI −0.45 to 0.07; *p* = 0.145).

Because the PSQI daytime dysfunction domain also showed a between-group imbalance at baseline (Table 2), it was likewise analyzed by baseline-adjusted ANCOVA. Both doses improved daytime dysfunction relative to placebo, with effects of similar magnitude (3 g/day, adjusted mean difference −0.30, 95% CI −0.60 to −0.005, *p* = 0.047; 6 g/day, −0.30, 95% CI −0.60 to −0.006, *p* = 0.046). These sleep endpoints were secondary and are interpreted as exploratory. The corresponding PSQI *p*-values are nominal and were not adjusted for multiplicity, and several effect estimates lay close to the null. No significant between-group differences were observed for the remaining PSQI components.

### 3.4. Exercise-Recovery Physiology: Blood Lactate Levels and Pharmacokinetics

Blood lactate levels were sampled pre-exercise, immediately post-exercise, and at 15 and 30 min of recovery for each treadmill test. At baseline, blood lactate increased from 1.09–1.20 mmol/L at rest to 2.22–2.75 mmol/L immediately after exercise across groups (mean increase, 1.36 mmol/L; values at or close to the commonly cited upper limit of the resting reference range, 2.0–2.4 mmol/L), although the response varied considerably among participants (57 of 119 remained below 2.0 mmol/L immediately after exercise); the lactate then declined to 1.61–1.79 mmol/L at 15 min and 1.32–1.36 mmol/L at 30 min of recovery (Table 5). In the FA set, the immediate post-exercise lactate was lower in the 6 g/day group than placebo at Week 8 (baseline-adjusted ANCOVA *p* = 0.027; unadjusted *p* = 0.033) and is reported descriptively alongside Cmax. Within the recovery time-point family (15 and 30 min, both doses), the 15 min lactate was significantly reduced at 6 g/day versus placebo (adjusted mean difference −0.39 mmol/L, 95% CI −0.61 to −0.17; *p* < 0.001, Holm-adjusted *p* = 0.002), whereas the 30 min difference was not significant. The 3 g/day group did not differ from placebo at any time point. Baseline and Week 8 values and the unadjusted changes from baseline at each time point are presented in Table 5.

Pharmacokinetic analysis of the serial lactate profiles was conducted to quantify overall exposure and identify the specific recovery phase affected (Figure 3). In baseline-adjusted ANCOVA, the primary exposure metric, total lactate AUC, was reduced in the 6 g/day group compared with placebo (adjusted mean difference −1278 mmol/L·s 95% CI −2197 to −359; *p* = 0.007), a finding that remained significant after Holm adjustment across the six contrasts of the pharmacokinetic family (adjusted *p* = 0.041). Cmax was also lower at 6 g/day (−0.35 mmol/L, 95% CI −0.65 to −0.04; *p* = 0.027), and post-peak AUC was reduced in the same direction (−579 mmol/L·s, 95% CI −1241 to 83; *p* = 0.086); neither remained significant after Holm adjustment (adjusted *p* = 0.137 and 0.221, respectively), so these phase-specific results are interpreted as exploratory. The unadjusted comparisons in Table 6 showed the same pattern (total AUC *p* = 0.015; post-peak AUC *p* = 0.032; Cmax *p* = 0.033). Pre-peak (production-phase) AUC and Tmax were unchanged; however, because no sample was taken during exercise or between 0 and 15 min of recovery, the decomposition cannot localize the reduction to the production or the recovery phase. The 3 g/day group did not differ from placebo on any pharmacokinetic index (all *p* ≥ 0.055). Detailed pharmacokinetic parameters for lactate are provided in Table 6, and these effects are visualized in Figure 3.

### 3.5. Safety and Tolerability

Safety was evaluated in the Safety Set (*n* = 120) at screening, baseline, and Weeks 4 and 8. No serious adverse events (SAEs) were reported. Between-group comparisons revealed no significant differences regarding the incidence, type, severity, or relatedness of adverse events (AEs) to the investigational product. At Week 4, two AEs (heartburn and palpitations) were reported in two participants in the 6 g/day group, while no events occurred in the 3 g/day or placebo groups (participants assessed at Week 4: *n* = 40, 40 and 39 for placebo, 3 g/day and 6 g/day). This difference was not statistically significant (6 g/day vs. placebo, 2/39 vs. 0/40; Fisher’s exact test *p* = 0.241). Both events were classified as mild and unrelated to the study product. Clinical safety monitoring (including hematology, biochemistry, lipid profiles, urinalysis, vital signs, and pregnancy testing) revealed no pre-specified clinically significant changes and no significant between-group differences across all study visits.

### 3.6. Exploratory Blood Markers: Cortisol Pharmacokinetics, NO, Glucose, and LDH

Blood biomarkers were analyzed in the FA set. Exercise-associated analytes (glucose, LDH, and cortisol) were sampled at time points relative to the treadmill challenge, whereas resting plasma nitrite was measured at baseline and Week 8. An isolated pharmacokinetic difference in cortisol Tmax was observed for the 6 g/day group versus placebo (*p* = 0.047); however, no other cortisol parameters differed between groups. Because this finding was isolated and not adjusted for multiplicity, it is considered exploratory. Resting plasma nitrite levels showed no significant between-group differences from baseline to Week 8 (Table 4). This finding is consistent with the arginine paradox interpretation, which suggests that clinical benefit may occur without a measurable rise in systemic NO. For glucose and LDH, fixed time-point comparisons and pharmacokinetic summaries consistently indicated no treatment effect at Week 8 (Table 5 and Table 6).

An isolated pre-exercise (resting) glucose difference was observed in the 6 g/day group (*p* = 0.032), whereas no post-exercise glucose parameters differed between groups. Similarly, an isolated LDH Tmax difference in the 3 g/day group (*p* = 0.009), unaccompanied by any change in AUC or Cmax, is considered exploratory.

### 3.7. Other Exploratory and Neutral Endpoints (Non-Blood Measures)

These exploratory endpoints were analyzed in the FA set. Neither the Multidimensional Fatigue Scale (MFS) nor the fatigue Visual Analogue Scale (VAS) demonstrated consistent between-group differences at Week 8 (Table 4). While within-group changes are provided in the tables for context, all inferences rely on the between-group contrasts.

## 4. Discussion

The most important finding of this study is that the effects of L-arginine differed across outcome domains and across dose. Confidence in this interpretation is supported first by the balance of baseline characteristics. The three randomized groups started without differences in most baseline characteristics, including demographics and anthropometrics, and the only exception was PSQI daytime dysfunction, for which both the 3 g/day and 6 g/day arms had significantly higher baseline values than placebo (*p* = 0.007 and *p* = 0.023, respectively), with 3 g/day numerically higher than 6 g/day but no statistically significant difference between the two active arms, and baseline cardiorespiratory fitness was within the normal range in all groups (Table 2). Importantly, this single imbalance was confined to the sleep domain rather than to the primary endpoint, and baseline FSS itself was balanced across groups (6 g/day versus placebo, 35.23 versus 34.23, *p* = 0.669). On this balanced footing, after 8 weeks of supplementation, only 6 g/day produced a significant improvement in the FSS relative to placebo (change −6.41 versus −2.23, *p* = 0.012), whereas 3 g/day changed essentially as placebo did (−2.30 versus −2.23, *p* = 0.935). Because this 6 g/day fatigue benefit retained significance after multiplicity adjustment within the fatigue family (Holm-adjusted *p* = 0.024), it is less likely to reflect chance alone, and, because the baseline FSS was balanced and the analysis was based on the change from baseline, it is also difficult to attribute this to the baseline imbalance alone. Nevertheless, the magnitude of this benefit should be interpreted with caution: the mean FSS score of the 6 g/day group at Week 8 (28.82) remained above the enrollment threshold of 27, indicating a reduction in fatigue relative to the placebo rather than its resolution.

The remaining endpoints consistently support this dose-differentiated pattern. Among the other fatigue measures, the 6 g/day group showed a CIS improvement in the same direction as the FSS but this did not survive multiplicity adjustment (nominal *p* = 0.029), and neither the MFS nor the VAS showed a clear between-group difference, so that several instruments pointed in the same direction while the strength of the signal weakened across scales. The same dose specificity was reproduced in the objective measure of post-exercise recovery, in which 6 g/day significantly reduced total lactate exposure (AUC) relative to placebo and this too was retained after multiplicity adjustment (baseline-adjusted *p* = 0.007, Holm-adjusted *p* = 0.041), with the difference most evident at 15 min of recovery (Holm-adjusted *p* = 0.002), whereas 3 g/day did not differ from placebo at any time point. In contrast, sleep showed a different dose pattern. In baseline-adjusted ANCOVA, both doses nominally improved the PSQI total score and daytime dysfunction relative to placebo (exploratory, unadjusted for multiplicity), whereas habitual sleep efficiency improved only at 3 g/day, and, although the total-score improvement was numerically larger at 3 g/day, the confidence intervals for the two doses overlapped and were not clearly distinguishable. Finally, the resting plasma nitrite showed no significant between-group change, which is in keeping with the arginine paradox, whereby functional changes can occur without a measurable rise in systemic NO [11,12].

The principal contribution of this trial lies in its focus on a subclinical, community-dwelling population. Prior evidence for L-arginine often stems from clinically defined disease cohorts (e.g., long COVID) [19], or multi-ingredient formulations in trained athletes, leaving single-ingredient L-arginine in non-diseased populations understudied [21]. By enrolling participants with everyday fatigue (FSS ≥ 27) and evaluating L-arginine as a single agent, this study addresses a critical evidence gap in this understudied population.

The various changes observed at 6 g/day are compatible with a blood-flow-based mechanism. Acute sleep deprivation impairs vascular endothelial function and alters hemodynamic and autonomic regulation in healthy adults [7], and L-arginine, as a substrate for eNOS, is known to promote endothelium-dependent vasodilation that redistributes blood flow to active muscle and assists oxygen delivery and metabolite clearance during and after exercise [8,9]. This mechanism would predict lower lactate values during recovery; the lower 15 min values at 6 g/day (Figure 3) are directionally consistent with that prediction, although, because the peak and early clearance phase were not sampled, the data cannot show that clearance was faster, and the phase-specific metrics did not survive multiplicity adjustment and this localization remains exploratory. Mechanistically, an improvement in fatigue would be expected to appear first in the aspects that interfere with physical activity and sustained function rather than in general tiredness, and the significant benefit of 6 g/day over placebo on the FSS was most apparent in the items reflecting interference with activity and function, which stood out in the same direction. The lactate findings pertain to peripheral recovery after a standardized exercise bout and do not by themselves explain everyday fatigue; whether the FSS changes and the recovery-phase lactate changes share a common perfusion-dependent mechanism cannot be determined from the present data; the lack of change in plasma nitrite may reflect the limitation of a short-lived systemic marker rather than a refutation of this mechanism.

In contrast, the precise reason that these changes did not appear at 3 g/day cannot be determined from the present data. In light of the mechanism described above, a possible explanation is that the functional dose response of L-arginine is not linear, so that a threshold dose is required to shift endothelial signaling and recovery-phase perfusion above habitual supply, and that in the present setting of a minimally impaired, community-dwelling population taking a single agent this threshold was higher, so that 3 g/day fell below it [11,12]. This is only an inference from the observed results, however, and does not directly establish the mechanism of the 3 g/day non-response. This will need to be clarified in follow-up studies that widen the dose range, measure perfusion, endothelial function, and muscle oxygenation directly, and account for individual differences such as aerobic capacity.

Consistent with the objective of our study, also reported, in an 8-week anti-fatigue trial of fermented porcine placenta extract, a significant improvement in the FSS total score relative to placebo, but the pattern of lactate change differed from ours. In their study, a nonsignificant tendency toward lower lactate was seen only in the production (pre-peak) AUC, with no significant change in the recovery (post-peak) AUC, whereas, in the present study, the reduction in lactate appeared to be concentrated in the recovery phase with the production phase unchanged [32]. Thus, although the FSS improved in both trials, the lactate phase accompanying that improvement was the opposite, and, in their study, the FSS improvement was dissociated from any recovery-phase lactate change. This difference is best explained by the differing mechanisms of the two test products. The anti-fatigue action of fermented placenta extract has been attributed to antioxidant (SOD and CAT) and anti-inflammatory (IL-1β and TNF-α) activity together with glycogen sparing, consistent with the action at the production and accumulation stage of the metabolic load. L-arginine, by contrast, acts as an eNOS substrate that promotes endothelium-dependent vasodilation and perfusion and thereby accelerates metabolite clearance in the recovery phase, so that its effect appearing in the recovery phase is mechanistically consistent. The same mechanistic contrast is reflected in the pattern of the FSS items. In the present study, the improvement was most pronounced in the items on feeling easily fatigued, fatigue preventing sustained physical activity, fatigue interfering with duties and responsibilities, and fatigue ranking among the three most disabling symptoms, whereas, in the study by Yoon and colleagues, it was most pronounced in the items on interference with physical functioning and reduced motivation, which can be interpreted as a difference grounded in the differing mechanisms of the two test products. The difference between the two studies is therefore better read not as a conflict but as a possible indication that different anti-fatigue substances act at different metabolic stages, which neither trial’s sampling frame can establish.

If the benefit of L-arginine is mediated through the eNOS-dependent improvement of recovery-phase perfusion, a reduction in lactate could reflect accelerated clearance, reduced production, or both. Hypothesis 2 was only partly supported: a modest between-group difference was observed at 15 min of recovery, whereas the peak and early clearance phase were not sampled. This inference should nonetheless be qualified by the nature of the analysis that supports it. The pre-/post-peak decomposition used to localize the lactate effect was exploratory rather than a protocol-registered procedure, so its inferences support rather than confirm the primary findings. Total lactate AUC, the principal exposure metric, remained significant after multiplicity adjustment (baseline-adjusted *p* = 0.007, Holm-adjusted *p* = 0.041), whereas the phase-specific metrics of post-peak AUC and Cmax were reduced in the same direction but did not survive adjustment and are treated as exploratory. A lower Cmax alone cannot distinguish accelerated clearance from reduced production; yet, the concurrent stability of the production-phase (pre-peak) AUC and Tmax would be compatible with a recovery-phase change, so the decomposition suggests rather than establishes that the reduction was localized to recovery. Prior reports that L-arginine attenuates exercise-induced lactate elevation in healthy men [37] and lowers blood lactate in athletes after chronic supplementation [38] predominantly emphasized production-phase effects, whereas our decomposition, following established lactate-kinetic modeling methods [24,25], is consistent with an additional recovery-phase contribution, and the two need not be mutually exclusive. The isolated cortisol Tmax difference at 6 g/day remains exploratory.

Resting blood lactate was within the normal range in all groups (1.1–1.2 mmol/L) [25], and immediate post-exercise values (2.2–2.8 mmol/L) were close to the commonly cited upper limit of the resting reference range (2.0–2.4 mmol/L). Lactate was therefore not examined as a cause of persistent everyday fatigue, but as an index of peripheral recovery assessed at 15 and 30 min after an identical standardized workload. Because lactate is a marker of exercise metabolism, we expected the kinetics of lactate and of lactate dehydrogenase (LDH) to move together; yet, in the present study, the 6 g/day effect appeared in lactate but not in LDH, so the two markers dissociated. This interpretation assumes that the exercise load produced measurable lactate accumulation; on average, blood lactate approximately doubled from rest to the end of exercise and declined during recovery (Table 5), although the response varied considerably among participants and nearly half remained below 2.0 mmol/L immediately after exercise. Whether lactate accumulates is determined relative to the individual lactate threshold rather than by an absolute percentage of VO_2_max [25], and the load used here, about 70% of VO_2_max sustained for 30 min, is expected to elicit a lactate response in most untrained adults, although the relative aerobic–anaerobic contribution at this fixed intensity differs among individuals. The dissociation of the two markers can be understood from the fact that they reflect different processes. Lactate has classically been regarded as a fatigue substance, but it is now understood as a metabolic fuel that is oxidized through the cell-to-cell lactate shuttle and contributes to ATP generation [25], so that the recovery-phase reduction in lactate may reflect the more efficient perfusion-dependent transport and oxidation of lactate rather than the simple removal of a waste product. In addition, the metabolic acidosis caused by the hydrogen ions (H^+^) that accumulate together with lactate arises largely from ATP hydrolysis and is handled by the bicarbonate buffer system, in which H^+^ + HCO_3_^−^ → H_2_CO_3_ → H_2_O + CO_2_, so that it is converted to carbon dioxide and removed by ventilation [39]. Both the transport and oxidation of lactate and the buffering and removal of H^+^ and CO_2_ depend on blood flow and gas exchange, that is, on perfusion. Blood LDH, by contrast, is a marker of the leakage of an intracellular enzyme and reflects muscle-cell membrane permeability and tissue turnover, a process separates from perfusion-dependent clearance. If L-arginine enhances perfusion through eNOS, the handling of lactate and the removal of H^+^ and CO_2_ would therefore be accelerated, producing a change in lactate kinetics, whereas LDH, which depends on membrane integrity, would remain unaffected, which coherently explains the dissociation between lactate and LDH. Although pH, bicarbonate, and carbon dioxide were not directly measured in the present study, the different trends of lactate and LDH relative to placebo across the immediate post-exercise and recovery time points in Table 5 are consistent with this interpretation and provide indirect support for it; the addition of future studies that include pH, bicarbonate, and ventilatory measures would allow clearer evidence attributable to L-arginine to be established.

The sleep endpoints showed a dose pattern different from that of fatigue. The baseline PSQI total score across the three groups was 6.10 to 6.74, above the cutoff of 5 and indicating mild sleep problems. In baseline-adjusted ANCOVA, sleep quality (total score) and daytime dysfunction improved significantly relative to placebo at both 3 g/day and 6 g/day without being dose-dependent, as shown in Figure 2, whereas habitual sleep efficiency improved from baseline at both doses but reached significance versus placebo only at 3 g/day. This pattern is better interpreted as a common effect of L-arginine, with overlapping confidence intervals for the two doses, than as a superiority of 3 g/day over 6 g/day. In particular, the fact that this sleep improvement occurred at both doses, whereas the fatigue benefit (FSS) was confined to 6 g/day, suggests that the sleep effect is independent of, rather than secondary to, the reduction in fatigue. It is also difficult to explain this effect by systemic nitric oxide alone, which showed no significant change owing to its short half-life.

Several mechanisms may underlie this common sleep effect. First, because oral L-arginine undergoes substantial first-pass metabolism that limits its systemic availability, 3 g/day and 6 g/day may have reached a similar systemic exposure for the sleep-related pathway, producing a common effect that was not proportional to dose. Second, L-arginine is known to increase growth hormone secretion mainly by suppressing endogenous somatostatin [40], and the GHRH and somatostatin system that regulates growth hormone secretion is also involved in the regulation of slow-wave sleep, with GHRH reported to promote non-REM (slow-wave) sleep [41]. However, in humans, this link is inconsistent and may differ by sex, and, because our sample was predominantly female and growth hormone was not directly measured, this pathway is offered as a hypothesis rather than a definitive explanation. Third, L-arginine promotes peripheral vasodilation by relaxing vascular smooth muscle through nitric oxide synthase [8,9], and the resulting distal heat loss is known to favor sleep onset, which may have contributed to the improvement in sleep. Because these mechanisms were not directly measured in the present study, in vivo follow-up studies that assess growth hormone secretion, ammonia excretion, and the hypothalamic-pituitary-adrenal (HPA) axis, together with polysomnography, will be needed to identify the causes of these findings more clearly.

In the present study, no serious adverse events were observed, and the regimen was well tolerated over 8 weeks, consistent with the known tolerability of oral L-arginine [42]. The two adverse events observed (heartburn and palpitations) both occurred in the 6 g/day group but were judged to be mild. Although L-arginine has raised safety concerns in a trial of patients with severe cardiac illness in the acute period after myocardial infarction [14], this concern is judged not to apply to the present study population of community-dwelling participants without known cardiovascular disease.

Our study was conducted in participants who were free of specific underlying disease but carried a potential risk of persistent stress, using a randomized, double-blind, placebo-controlled design, and it evaluated patient-reported outcomes together with standardized exercise and metabolic testing. Several limitations should be considered. First, the sample size (40 participants per group) was small. It was calculated a priori for the comparison of each active arm with placebo on the primary endpoint; the secondary and mechanistic endpoints and the comparison between the two doses were therefore underpowered and should be interpreted as exploratory, and the observed between-group difference in FSS (4.18 points) was smaller than the 5-point difference assumed in the sample size calculation, limiting the precision of the effect estimate. The observed between-group difference in FSS change (4.18 points; pooled SD ≈ 7.1) may serve as a reference effect size for future trials: detecting a difference of this magnitude with 80% power would require approximately 46 participants per group for a single comparison, or about 55 per group when two active arms are compared with placebo, before allowance for attrition. Future trials should also include blood sampling at 3–8 min after exercise, a reassessment of VO_2_max at the end of the intervention, objective sleep measures, and a sex-balanced sample. Although tolerability was a stated objective, a sample of 40 per group cannot detect uncommon adverse events. In addition, the multiplicity adjustment, the presentation of the FA Set as the primary analysis population, and several baseline-adjusted analyses were applied post hoc, and the study was conducted at a single center. Second, the participants were relatively homogeneous and predominantly female (about 79%), which is likely to limit the general applicability of the findings; in particular, because lactate and LDH responses reflect physiological characteristics of skeletal muscle such as fiber-type composition and glycolytic capacity, and these characteristics differ between the sexes [43], the metabolic findings of this study should be confirmed in sex-balanced samples. Third, a contribution from residual baseline imbalances cannot be excluded, as the 3 g/day arm had, on average, a lower baseline aerobic fitness (VO_2_max) and a higher baseline FSS than the other arms, and aerobic fitness was not entered as a covariate in the efficacy analyses. Fourth, blood lactate was sampled before exercise, immediately after exercise, and at 15 and 30 min of recovery, following the sampling frame of a previous trial in a comparable population [32]. This frame did not include the 3–8-min post-exercise window in which blood lactate typically peaks, as lactate continues to efflux from muscle into blood after exercise cessation [25]. The time to peak depends on exercise intensity and on the muscle-to-blood lactate gradient, being shorter after lower-intensity than after supramaximal exercise [44], and it also varies among individuals with the relative anaerobic contribution at a given relative intensity, which is determined by fiber-type composition, lactate transporter expression, and training status [45,46]. In the present sample, the immediate post-exercise value was the highest observed value in most participants, but, because no sample was taken between 0 and 15 min, a higher transient peak cannot be excluded in participants with a greater anaerobic contribution, and the immediate post-exercise value may therefore underestimate the true peak. The lactate response to the standardized load also varied considerably among participants, and a clinically meaningful threshold for the observed lactate changes has not been established. Accordingly, the immediate post-exercise value is reported only as the value at that time point, no conclusion is drawn about peak lactate or early clearance rate, and the lactate findings are confined to the 15- and 30-min recovery comparisons and interpreted as supportive mechanistic evidence rather than as an efficacy outcome. Fifth, VO_2_max was measured only at screening; because it was not reassessed at Week 8, a change in aerobic fitness cannot be completely excluded, and any effect of L-arginine on maximal aerobic capacity could not be determined. Sixth, the primary fatigue endpoint and the sleep endpoints were patient-reported and therefore subject to recall and expectancy biases, and no objective sleep measure (e.g., actigraphy or polysomnography) was included; in addition, the mean FSS score of the 6 g/day group at Week 8 remained above the enrollment threshold; because a minimal clinically important difference for the FSS has not been established in non-clinical community populations, the clinical relevance of the observed reduction should be interpreted with caution. These limitations should be addressed in adequately powered multicenter studies with a longer follow-up that account for sex balance and aerobic fitness, and, if the direct vascular, endothelial-function, and muscle-oxygenation measures noted above are also included, the causes of L-arginine-related post-exercise fatigue recovery and multidimensional fatigue improvement, and of the non-dose-proportional pattern of these effects, could be more clearly elucidated in individuals at potential risk of persistent stress.

## 5. Conclusions

As one of the first randomized, placebo-controlled trials of single-ingredient L-arginine in community-dwelling adults with persistent subclinical fatigue, this study demonstrates that an 8-week regimen was well-tolerated. Compared with placebo, 6 g/day of L-arginine produced a significant reduction in the primary fatigue outcome (FSS) after multiplicity adjustment, accompanied by supportive improvements in the CIS score that did not survive multiplicity adjustment and a modest reduction in total lactate exposure (AUC) and in 15-min recovery lactate, which remained within the normal range and whose clinical relevance is not established. At the end of the 8-week intervention, the mean FSS score of the 6 g/day group (28.82) remained above the enrollment threshold of 27; on average, participants therefore still met the study definition of persistent fatigue after treatment, and the intervention did not bring participants below the enrollment threshold on average. The present findings indicate a reduction in fatigue severity relative to the placebo and should not be interpreted as showing that persistent fatigue was resolved. In an exploratory decomposition, production-phase indices (pre-peak AUC and Tmax) were unchanged while the 15-min recovery lactate was significantly lower (Holm-adjusted *p* = 0.002); however, the phase-specific pharmacokinetic metrics (post-peak AUC and Cmax) did not survive multiplicity adjustment, the 3–8-min peak and the early clearance phase were not sampled, and whether the reduction reflects accelerated clearance, reduced production, or altered perfusion cannot be determined from these data. The absence of changes in plasma nitrite is consistent with the arginine paradox. In addition, in exploratory baseline-adjusted analyses, sleep quality (PSQI total score) and daytime dysfunction showed nominal improvements relative to the placebo at both doses, a common, non-dose-dependent pattern that contrasts with the fatigue benefit confined to 6 g/day (habitual sleep efficiency reached nominal significance only at 3 g/day). Given the modest sample size, this secondary finding warrants confirmation in larger trials. Collectively, these results suggest that 6 g/day of L-arginine reduced fatigue relative to placebo and is a candidate regimen warranting confirmatory trials for managing persistent fatigue, potentially serving as an adjunct to the standard workup for reversible fatigue-related factors.

## Figures and Tables

**Table 1:** Study product adherence (FA Set).

Adherence Variable	Placebo (*n* = 40)	L-Arginine		*p*-Value *	*p*-Value **	*p*-Value ^†^
		3 g/day (*n* = 40)	6 g/day (*n* = 39)			
Compliance at Week 4, % (mean ± SD)	90.85 ± 7.22	93.24 ± 5.72	89.38 ± 6.37	0.104	0.342	0.006
Compliance at Week 8, % (mean ± SD)	91.08 ± 8.03	92.51 ± 8.56	92.21 ± 6.42	0.454	0.498	0.866

Note: Data are mean ± SD. Analysis set: full analysis (FA) set (*n* = 119); Week 8 compliance was available for participants who attended Visit 4 (placebo *n* = 39, 3 g/day *n* = 38, 6 g/day *n* = 38). Compliance (%) = (sachets taken/planned) × 100. An average intake < 80% was a prespecified criterion for discontinuation and exclusion from the PP set; no participant fell below this threshold. *p*-value with * or ** is independent *t*-test compared between Placebo and L-arginine 3 g/day or 6 g/day, respectively. ^†^ *p*-value for the comparison between L-arginine 3 g/day and 6 g/day (independent *t*-test).

**Table 2:** Baseline characteristics (FA Set).

Characteristics (Mean ± SD)	Oral L-Arginine			Placebo vs. 3 g/day *p*-Value	Placebo vs. 6 g/day *p*-Value	3 g/day vs. 6 g/day *p*-Value
	Placebo (*n* = 40)	3 g/day (*n* = 40)	6 g/day (*n* = 39)			
Demographics and anthropometrics						
Age, years	44.20 ± 7.09	43.88 ± 7.77	44.05 ± 6.82	0.846 ^a^	0.925 ^a^	0.915 ^a^
Female, *n* (%)	30 (75.0)	33 (82.5)	31 (79.5)	0.412 ^c^	0.635 ^c^	0.733 ^c^
Height, cm	164.56 ± 9.00	163.25 ± 8.20	165.29 ± 7.79	0.498 ^a^	0.698 ^a^	0.259 ^a^
Weight, kg	65.02 ± 11.26	61.77 ± 11.67	65.55 ± 14.44	0.209 ^a^	0.854 ^a^	0.204 ^a^
BMI, kg/m^2^	23.91 ± 2.96	23.06 ± 3.00	23.81 ± 3.87	0.203 ^a^	0.893 ^a^	0.338 ^a^
Clinical baselines (fatigue and sleep)						
FSS total (primary)	34.23 ± 6.34	36.60 ± 7.44	35.23 ± 7.45	0.129 ^b^	0.669 ^b^	0.340 ^b^
CIS total	67.50 ± 12.96	70.95 ± 12.88	70.77 ± 13.64	0.202 ^b^	0.278 ^a^	0.688 ^b^
MFS total	74.63 ± 19.84	78.85 ± 18.09	76.18 ± 19.71	0.323 ^a^	0.576 ^b^	0.837 ^b^
Fatigue VAS, mm	47.90 ± 17.86	49.50 ± 19.34	48.97 ± 21.86	0.647 ^b^	0.811 ^a^	0.866 ^b^
PSQI total	6.10 ± 2.61	6.45 ± 2.63	6.74 ± 3.05	0.573 ^b^	0.446 ^b^	0.805 ^b^
Selected PSQI domains						
Subjective sleep quality, score	1.30 ± 0.69	1.30 ± 0.61	1.44 ± 0.60	0.957 ^b^	0.391 ^b^	0.401 ^b^
Habitual sleep efficiency, score	0.25 ± 0.59	0.43 ± 0.78	0.33 ± 0.74	0.276 ^b^	0.698 ^b^	0.495 ^b^
Daytime dysfunction, score	1.05 ± 0.85	1.55 ± 0.75	1.49 ± 0.79	0.007 ^b^	0.023 ^b^	0.600 ^b^
Exercise capacity and biomarker baselines						
VO_2_max (mL/kg/min)	31.99 ± 4.05	30.73 ± 3.64	31.54 ± 4.30	0.147 ^a^	0.633 ^a^	0.367 ^a^
Total physical activity (GPAQ), METs-min/week	2831.50 ± 3421.95	3065.50 ± 2850.72	2971.79 ± 2974.12	0.590 ^b^	0.566 ^b^	0.867 ^b^
Lactate, pre-exercise (mmol/L)	1.10 ± 0.48	1.20 ± 0.68	1.09 ± 0.50	0.840 ^b^	0.871 ^b^	0.720 ^b^
Glucose, pre-exercise (mg/dL)	96.65 ± 14.34	93.13 ± 8.88	96.56 ± 11.05	0.320 ^b^	0.379 ^b^	0.075 ^b^
LDH, pre-exercise (U/L)	180.63 ± 42.83	175.70 ± 50.73	172.77 ± 32.91	0.133 ^b^	0.495 ^b^	0.586 ^b^
Cortisol, pre-exercise (µg/dL)	8.62 ± 3.59	9.34 ± 3.63	9.21 ± 4.14	0.381 ^a^	0.787 ^b^	0.573 ^b^
Nitric oxide (µmol/L)	0.38 ± 0.42	0.34 ± 0.42	0.27 ± 0.40	0.488 ^b^	0.076 ^b^	0.388 ^b^

*p*-values are between-group comparisons (Placebo vs. 3 g/day, Placebo vs. 6 g/day, and 3 g/day vs. 6 g/day): ^a^ independent *t*-test; ^b^ Mann–Whitney U test; ^c^ Chi-square test. Total physical activity was assessed with the Global Physical Activity Questionnaire (GPAQ) and is expressed as metabolic equivalent of task minutes per week (METs-min/week); changes during the intervention are presented in Supplementary Table S5 (PP set).

**Table 3:** Between-group differences in change from baseline in Fatigue Severity Scale (FSS) scores (FA Set).

		L-Arginine			Placebo vs. 3 g/day *p*-Value	Placebo vs. 6 g/day *p*-Value
		Placebo (*n* = 40)	3 g/day (*n* = 40)	6 g/day (*n* = 39)		
FSS total score	Δ Week 4	−1.18 ± 3.68	−1.15 ± 5.01	−3.87 ± 4.62	0.980 ^a^	0.006 ^b^
	Δ Week 8	−2.23 ± 6.02	−2.30 ± 6.85	−6.41 ± 8.02	0.935 ^b^	0.012 ^b^
FSS individual items (changed from baseline, Δ Week 8)						
Motivation lower when fatigued		−0.60 ± 1.37	−0.65 ± 1.19	−0.87 ± 1.61	0.703 ^b^	0.187 ^b^
Exercise brings on fatigue		−0.55 ± 1.50	−0.23 ± 1.25	−0.44 ± 1.62	0.491 ^b^	0.837 ^b^
Easily fatigued		−0.40 ± 1.28	−0.30 ± 1.09	−1.00 ± 1.67	0.936 ^b^	0.005 ^b^
Fatigue interferes with physical functioning		−0.45 ± 1.43	−0.68 ± 0.97	−0.87 ± 1.22	0.186 ^b^	0.100 ^b^
Fatigue causes frequent problems		−0.33 ± 1.05	−0.20 ± 1.32	−0.67 ± 1.38	0.753 ^b^	0.159 ^b^
Fatigue prevents sustained physical activity		0.03 ± 1.05	0.15 ± 1.42	−0.67 ± 1.15	0.698 ^b^	0.008 ^b^
Fatigue interferes with duties/responsibilities		0.20 ± 1.07	0.03 ± 1.14	−0.54 ± 1.31	0.569 ^b^	0.009 ^b^
Fatigue among 3 most disabling symptoms		0.00 ± 1.32	−0.28 ± 1.26	−0.69 ± 1.26	0.271 ^b^	0.041 ^b^
Fatigue interferes with work/family/social life		−0.13 ± 1.34	−0.15 ± 1.14	−0.67 ± 0.98	0.988 ^b^	0.062 ^b^

*p*-values are between-group comparisons versus placebo (Placebo vs. 3 g/day and Placebo vs. 6 g/day): ^a^ independent *t*-test; ^b^ Mann–Whitney U test. Δ, change from baseline (Week 4 or Week 8 value − baseline value)”.

**Table 4:** Between-group differences in change from baseline in CIS, MFS, fatigue VAS, PSQI total scores, and nitric oxide (NO) (FA Set).

		Oral L-Arginine			Placebo vs. 3 g/day *p*-Value	Placebo vs. 6 g/day *p*-Value
		Placebo (*n* = 40)	3 g/day (*n* = 40)	6 g/day (*n* = 39)		
CIS total	Δ Week 4	−2.13 ± 10.95	−4.65 ± 11.76	−5.97 ± 12.28	0.323 ^a^	0.126 ^b^
	Δ Week 8	−0.78 ± 11.56	−5.43 ± 10.55	−7.44 ± 12.18	0.064 ^a^	0.029 ^b^
MFS total	Δ Week 4	−2.85 ± 12.93	−0.45 ± 15.18	−3.00 ± 11.71	0.449 ^a^	0.957 ^a^
	Δ Week 8	−3.33 ± 15.11	−4.55 ± 14.20	−6.08 ± 13.55	0.710 ^a^	0.776 ^b^
Fatigue Visual Analogue Scale (VAS) (mm)	Δ Week 4	−2.65 ± 22.02	−7.50 ± 18.78	−6.41 ± 16.14	0.111 ^b^	0.287 ^b^
	Δ Week 8	−7.40 ± 19.13	−10.50 ± 23.42	−10.26 ± 22.18	0.519 ^a^	0.541 ^a^
PSQI total	Δ Week 8	0.03 ± 2.34	−1.33 ± 1.90	−1.15 ± 2.61	0.009 ^b^	0.097 ^b^
NO (µmol/L)	Δ Week 8	−0.04 ± 0.49	−0.15 ± 0.55	−0.01 ± 0.55	0.192 ^b^	0.776 ^b^

*p*-values are between-group comparisons versus placebo (Placebo vs. 3 g/day and Placebo vs. 6 g/day): ^a^ independent *t*-test; ^b^ Mann–Whitney U test. Δ, change from baseline (Week 4 or Week 8 value − baseline value)”.

**Table 5:** Exercise-recovery variables at baseline and Week 8 and change from baseline (Δ Week 8) (FA Set).

			Oral L-Arginine			Placebo vs. 3 g/day *p*-Value	Placebo vs. 6 g/day *p*-Value
			Placebo (*n* = 40)	3 g/day (*n* = 40)	6 g/day (*n* = 39)		
Lactate (mmol/L)	Before exercise	Baseline	1.10 ± 0.48	1.20 ± 0.68	1.09 ± 0.50	0.840 ^b^	0.871 ^b^
		Week 8	1.24 ± 0.61	1.13 ± 0.47	1.08 ± 0.57		
		Change	0.14 ± 0.57	−0.08 ± 0.54	−0.01 ± 0.67	0.373 ^b^	0.566 ^b^
	Immediately post-exercise	Baseline	2.50 ± 1.09	2.22 ± 1.34	2.75 ± 1.43	0.050 ^b^	0.573 ^b^
		Week 8	2.35 ± 1.08	1.90 ± 1.09	2.16 ± 1.04		
		Change	−0.15 ± 0.85	−0.31 ± 0.68	−0.59 ± 0.94	0.324 ^b^	0.033 ^a^
	15 min recovery	Baseline	1.63 ± 0.62	1.61 ± 0.81	1.79 ± 0.86	0.250 ^b^	0.735 ^b^
		Week 8	1.64 ± 0.75	1.48 ± 0.74	1.35 ± 0.57		
		Change	0.01 ± 0.61	−0.13 ± 0.48	−0.44 ± 0.59	0.272 ^a^	0.002 ^b^
	30 min recovery	Baseline	1.36 ± 0.47	1.32 ± 0.61	1.32 ± 0.54	0.231 ^b^	0.222 ^b^
		Week 8	1.40 ± 0.63	1.21 ± 0.43	1.25 ± 0.55		
		Change	0.03 ± 0.51	−0.11 ± 0.42	−0.07 ± 0.50	0.707 ^b^	0.614 ^b^
Glucose (mg/dL)	Before exercise	Baseline	96.65 ± 14.34	93.13 ± 8.88	96.56 ± 11.05	0.320 ^b^	0.379 ^b^
		Week 8	100.03 ± 17.14	94.10 ± 7.14	94.46 ± 9.22		
		Change	3.38 ± 8.78	0.97 ± 6.80	−2.10 ± 10.96	0.272 ^b^	0.032 ^b^
	Immediately post-exercise	Baseline	99.13 ± 16.09	96.35 ± 10.54	97.87 ± 10.17	0.531 ^b^	0.810 ^b^
		Week 8	98.83 ± 14.69	95.35 ± 10.26	95.54 ± 9.18		
		Change	−0.30 ± 8.72	−1.00 ± 6.55	−2.33 ± 9.25	0.686 ^a^	0.603 ^b^
	15 min recovery	Baseline	96.90 ± 15.89	92.63 ± 13.43	93.10 ± 9.72	0.116 ^b^	0.441 ^b^
		Week 8	96.73 ± 16.51	92.85 ± 10.66	94.28 ± 10.96		
		Change	−0.18 ± 12.08	0.22 ± 10.51	1.18 ± 9.37	0.870 ^b^	0.798 ^b^
	30 min recovery	Baseline	94.58 ± 15.56	90.40 ± 14.68	91.33 ± 9.88	0.110 ^b^	0.468 ^b^
		Week 8	95.80 ± 17.28	90.58 ± 9.63	92.31 ± 9.61		
		Change	1.22 ± 12.39	0.17 ± 11.32	0.97 ± 7.74	0.464 ^b^	0.421 ^b^
Lactate dehydrogenase, LDH (U/L)	Before exercise	Baseline	180.63 ± 42.83	175.70 ± 50.73	172.77 ± 32.91	0.133 ^b^	0.495 ^b^
		Week 8	184.28 ± 33.75	168.78 ± 31.10	194.26 ± 73.24		
		Change	3.65 ± 31.26	−6.92 ± 33.87	21.49 ± 74.02	0.110 ^b^	0.739 ^b^
	Immediately post-exercise	Baseline	197.13 ± 53.19	181.95 ± 35.84	188.18 ± 39.11	0.229 ^b^	0.720 ^b^
		Week 8	191.15 ± 46.29	174.48 ± 28.65	189.00 ± 58.75		
		Change	−5.97 ± 64.30	−7.47 ± 23.53	0.82 ± 64.16	0.620 ^b^	0.761 ^b^
	15 min recovery	Baseline	191.25 ± 64.60	181.80 ± 45.52	180.49 ± 30.90	0.178 ^b^	0.662 ^b^
		Week 8	185.83 ± 46.56	176.95 ± 29.37	176.00 ± 29.04		
		Change	−5.43 ± 64.82	−4.85 ± 38.98	−4.49 ± 25.60	0.711 ^b^	0.341 ^b^
	30 min recovery	Baseline	181.70 ± 33.37	185.15 ± 62.20	179.46 ± 27.48	0.535 ^b^	0.891 ^b^
		Week 8	179.35 ± 26.26	182.38 ± 60.72	175.00 ± 26.27		
		Change	−2.35 ± 23.38	−2.78 ± 24.88	−4.46 ± 19.24	0.795 ^b^	0.447 ^b^
Cortisol (µg/dL)	Before exercise	Baseline	8.62 ± 3.59	9.34 ± 3.63	9.21 ± 4.14	0.381 ^a^	0.787 ^b^
		Week 8	9.78 ± 4.24	9.80 ± 3.47	10.09 ± 4.40		
		Change	1.16 ± 4.08	0.47 ± 2.76	0.88 ± 3.06	0.378 ^a^	0.733 ^a^
	Immediately post-exercise	Baseline	8.32 ± 3.53	9.64 ± 5.35	9.65 ± 4.39	0.419 ^b^	0.165 ^b^
		Week 8	8.81 ± 3.56	9.55 ± 3.84	9.04 ± 4.23		
		Change	0.49 ± 3.66	−0.09 ± 3.69	−0.61 ± 4.10	0.711 ^b^	0.108 ^b^
	15 min recovery	Baseline	7.85 ± 3.32	9.03 ± 5.05	9.19 ± 4.32	0.516 ^b^	0.158 ^b^
		Week 8	8.44 ± 3.51	8.88 ± 3.55	8.48 ± 4.14		
		Change	0.60 ± 3.48	−0.16 ± 3.53	−0.71 ± 4.32	0.583 ^b^	0.075 ^b^
	30 min recovery	Baseline	6.82 ± 2.72	8.06 ± 4.33	8.24 ± 4.14	0.294 ^b^	0.141 ^b^
		Week 8	7.46 ± 3.05	8.02 ± 3.06	7.91 ± 4.00		
		Change	0.64 ± 2.91	−0.04 ± 2.89	−0.33 ± 4.55	0.297 ^a^	0.097 ^b^

*p*-values are between-group comparisons versus placebo (Placebo vs. 3 g/day and Placebo vs. 6 g/day): ^a^ independent *t*-test; ^b^ Mann–Whitney U test. *p*-values in the Baseline rows compare baseline values between groups; *p*-values in the Change rows compare unadjusted changes from baseline (Week 8 − baseline). Values are for the FA set; inferential statements for lactate in the text use baseline-adjusted ANCOVA with Holm adjustment (15 min recovery, 6 g/day: ANCOVA *p* < 0.001, Holm-adjusted *p* = 0.002).

**Table 6:** Exercise-recovery pharmacokinetics (PK) parameters: change from baseline at Week 8 (FA Set).

		Oral L-Arginine			Placebo vs. 3 g/day *p*-Value	Placebo vs. 6 g/day *p*-Value
		Placebo (*n* = 40)	3 g/day (*n* = 40)	6 g/day (*n* = 39)		
Lactate	Total AUC (mmol/L·s	−24.52 ± 2454.41	−747.07 ± 1984.52	−1487.48 ± 2592.57	0.176 ^b^	0.015 ^b^
	Pre-peak AUC (mmol/L·s)	−0.20 ± 1760.49	−275.14 ± 1901.00	−773.28 ± 2049.52	0.504 ^a^	0.170 ^b^
	Post-peak AUC (mmol/L·s)	−5.56 ± 1844.88	−471.94 ± 1953.21	−714.20 ± 1538.98	0.590 ^b^	0.032 ^b^
	Cmax (mmol/L)	−0.08 ± 0.88	−0.31 ± 0.69	−0.53 ± 0.93	0.198 ^a^	0.033 ^a^
	Tmax (s)	129.29 ± 1078.81	109.33 ± 1224.00	187.82 ± 1000.99	0.419 ^b^	0.803 ^b^
Glucose	Total AUC (mg/dL·s)	6197.34 ± 36,822.10	1764.89 ± 27,911.12	−4258.58 ± 31,225.12	0.422 ^b^	0.151 ^b^
	Cmax (mg/dL)	−1.25 ± 12.05	−0.13 ± 11.40	−2.28 ± 7.85	0.521 ^b^	0.817 ^b^
	Tmax (s)	−637.38 ± 1639.30	−561.18 ± 1708.97	291.74 ± 1992.57	0.779 ^b^	0.169 ^b^
LDH	Total AUC (U/L·s)	−9263.45 ± 150,542.55	−23,123.60 ± 100,497.81	26,270.86 ± 177,380.63	0.433 ^b^	0.949 ^b^
	Cmax (U/L)	−5.40 ± 75.89	−13.13 ± 47.76	13.77 ± 95.44	0.464 ^b^	0.576 ^b^
	Tmax (s)	−551.33 ± 1645.73	381.25 ± 1538.26	−828.87 ± 1935.29	0.009 ^b^	0.494 ^a^
Cortisol	Total AUC (µg/dL·s)	3360.73 ± 14,640.86	469.11 ± 12,291.00	−599.36 ± 16,112.56	0.342 ^a^	0.266 ^b^
	Cmax (µg/dL)	1.22 ± 3.81	0.23 ± 3.43	−0.06 ± 3.86	0.225 ^a^	0.176 ^b^
	Tmax (s)	60.78 ± 1784.30	39.80 ± 1649.95	−693.28 ± 1834.44	0.446 ^b^	0.047 ^b^

*p*-values are between-group comparisons versus placebo (Placebo vs. 3 g/day and Placebo vs. 6 g/day): ^a^ independent *t*-test; ^b^ Mann–Whitney U test. Values are unadjusted changes from baseline; inferential statements for lactate in the text use baseline-adjusted ANCOVA with Holm adjustment across six contrasts (6 g/day: total AUC *p* = 0.007, Holm-adjusted *p* = 0.041; post-peak AUC *p* = 0.086; Cmax *p* = 0.027; 3 g/day: all *p* ≥ 0.055).

## Data Availability

The original contributions presented in the study are included in the article/Supplementary Materials. Further inquiries for any data presented in the study can be directed to the corresponding authors.

## Supplementary Materials

The following supporting information can be downloaded at: https://www.mdpi.com/article/10.3390/nu18203375/s1, Table S1: Consolidated schedule for this study; Table S2: Medical and medication history; Table S3: Lifestyle factors (smoking and alcohol); Table S4: Summary of dietary intake (PP set); Table S5: Summary of total physical activities (METs/week, PP set); Table S6: Results of the base value difference for the validity evaluation variable (PP set); Table S7: Results of FSS questionnaire analysis (PP set); Table S8: Per-protocol sensitivity analyses of the secondary fatigue, sleep, and blood lactate endpoints (PP set).

## Abbreviations

The following abbreviations are used in this manuscript:

ALT	Alanine aminotransferase
ANCOVA	Analysis of covariance
AST	Aspartate aminotransferase
AUC	Area-under-the-curve
BMI	Body mass index
CIS	Checklist individual strength
Cmax	Maximum observed concentration
CRF	Case report form
e-CRFs	Electronic case report forms
EDC	Electronic data capture
eNOS	endothelial NO synthase
FA	Full analysis
FSS	Fatigue Severity Scale
IPs	Investigational products
IRB	Institutional Review Board
LDH	Lactate dehydrogenase
MFS	Multidimensional fatigue scale
NO	Nitric oxide or nitric-oxide
PP	Per-protocol
PSQI	Pittsburgh Sleep Quality Index
PSQI-K	Korean Pittsburgh Sleep Quality Index
RPE	Rating of perceived exertion
SAEs	Serious adverse events
SBP	Systolic blood pressure
Tmax	Time of maximum concentration
ULN	Upper limit of normal
χ^2^	Chi-square test

## References

[1] Kim C.H., Shin H.C., Won C.W. (2005). Prevalence of chronic fatigue and chronic fatigue syndrome in Korea: Community-based primary care study. J. Korean Med. Sci..

[2] Kim M. (2023). OECD Average Annual Hours Worked: Comparative Analysis and Implications.

[3] Lim E.-J., Lee J.-S., Lee E.-J., Jeong S.-J., Park H.-Y., Ahn Y.-C., Son C.-G. (2021). Nationwide epidemiological characteristics of chronic fatigue syndrome in South Korea. J. Transl. Med..

[4] Lee O., Park S., Kim Y., So W.-Y. (2022). Participation in sports activities before and after the outbreak of COVID-19: Analysis of data from the 2020 Korea National Sports Participation Survey. Healthcare.

[5] Böger R.H. (2004). Asymmetric dimethylarginine, an endogenous inhibitor of nitric oxide synthase, explains the “L-arginine paradox” and acts as a novel cardiovascular risk factor. J. Nutr..

[6] Ghiadoni L., Donald A.E., Cropley M., Mullen M.J., Oakley G., Taylor M., O’Connor G., Betteridge J., Klein N., Steptoe A. (2000). Mental stress induces transient endothelial dysfunction in humans. Circulation.

[7] Sauvet F., Leftheriotis G., Gomez-Merino D., Langrume C., Drogou C., Van Beers P., Bourrilhon C., Florence G., Chennaoui M. (2010). Effect of acute sleep deprivation on vascular function in healthy subjects. J. Appl. Physiol..

[8] Förstermann U., Sessa W.C. (2012). Nitric oxide synthases: Regulation and function. Eur. Heart J..

[9] Siasos G., Tousoulis D., Antoniades C., Stefanadi E., Stefanadis C. (2007). L-Arginine, the substrate for NO synthesis: An alternative treatment for premature atherosclerosis?. Int. J. Cardiol..

[10] Khalaf D., Krüger M., Wehland M., Infanger M., Grimm D. (2019). The effects of oral L-arginine and L-citrulline supplementation on blood pressure. Nutrients.

[11] Kurz S., Harrison D. (1997). Insulin and the arginine paradox. J. Clin. Investig..

[12] Tentolouris C., Tousoulis D., Stefanadis C. (2004). L-arginine “paradox” in coronary atherosclerosis. Circulation.

[13] Bode-Böger S.M., Muke J., Surdacki A., Brabant G., Böger R.H., Frölich J.C. (2003). Oral L-arginine improves endothelial function in healthy individuals older than 70 years. Vasc. Med..

[14] Schulman S.P., Becker L.C., Kass D.A., Champion H.C., Terrin M.L., Forman S., Ernst K.V., Kelemen M.D., Townsend S.N., Capriotti A. (2006). L-arginine therapy in acute myocardial infarction: The Vascular Interaction with Age in Myocardial Infarction (VINTAGE MI) randomized clinical trial. JAMA.

[15] Walker H.A., McGing E., Fisher I., Böger R.H., Bode-Böger S.M., Jackson G., Ritter J.M., Chowienczyk P.J. (2001). Endothelium-dependent vasodilation is independent of the plasma L-arginine/ADMA ratio in men with stable angina: Lack of effect of oral L-arginine on endothelial function, oxidative stress and exercise performance. J. Am. Coll. Cardiol..

[16] Bailey S.J., Winyard P.G., Vanhatalo A., Blackwell J.R., DiMenna F.J., Wilkerson D.P., Jones A.M. (2010). Acute L-arginine supplementation reduces the O_2_ cost of moderate-intensity exercise and enhances high-intensity exercise tolerance. J. Appl. Physiol..

[17] Rezaei S., Gholamalizadeh M., Tabrizi R., Nowrouzi-Sohrabi P., Rastgoo S., Doaei S. (2021). The effect of L-arginine supplementation on maximal oxygen uptake: A systematic review and meta-analysis. Physiol. Rep..

[18] Viribay A., Burgos J., Fernández-Landa J., Seco-Calvo J., Mielgo-Ayuso J. (2020). Effects of arginine supplementation on athletic performance based on energy metabolism: A systematic review and meta-analysis. Nutrients.

[19] Tosato M., Calvani R., Picca A., Ciciarello F., Galluzzo V., Coelho-Júnior H.J., Di Giorgio A., Di Mario C., Gervasoni J., Gremese E. (2022). Effects of L-arginine plus vitamin C supplementation on physical performance, endothelial function, and persistent fatigue in adults with long COVID: A single-blind randomized controlled trial. Nutrients.

[20] Krupp L.B., LaRocca N.G., Muir-Nash J., Steinberg A.D. (1989). The fatigue severity scale: Application to patients with multiple sclerosis and systemic lupus erythematosus. Arch. Neurol..

[21] Motta B.A., de Arruda L.K., Zanella P.B. (2025). Is isolated arginine supplementation an effective ergogenic aid for athletes and physically active individuals? A systematic review of randomized clinical trials. Sport Sci. Health.

[22] Worm-Smeitink M., Gielissen M., Bloot L., Van Laarhoven H., Van Engelen B., Van Riel P., Bleijenberg G., Nikolaus S., Knoop H. (2017). The assessment of fatigue: Psychometric qualities and norms for the Checklist Individual Strength. J. Psychosom. Res..

[23] Buysse D.J., Reynolds C.F., Monk T.H., Berman S.R., Kupfer D.J. (1989). The Pittsburgh Sleep Quality Index: A new instrument for psychiatric practice and research. Psychiatry Res..

[24] Durand R., Galli M., Chenavard M., Bandiera D., Freund H., Messonnier L.A. (2021). Modelling of blood lactate time-courses during exercise and/or the subsequent recovery: Limitations and few perspectives. Front. Physiol..

[25] Goodwin M.L., Harris J.E., Hernández A., Gladden L.B. (2007). Blood lactate measurements and analysis during exercise: A guide for clinicians. J. Diabetes Sci. Technol..

[26] Park S., Lee S., Lee Y., Lee M. (2022). Normative reference values of physical fitness levels in Koreans: Results from the National Fitness Award Project (2017–2019). Exerc. Sci..

[27] Chung K.-I., Song C.-H. (2001). Clinical usefulness of fatigue severity scale for patients with fatigue, and anxiety or depression. Korean J. Psychosom. Med..

[28] Vercoulen J.H., Swanink C.M., Fennis J.F., Galama J.M., van der Meer J.W., Bleijenberg G. (1994). Dimensional assessment of chronic fatigue syndrome. J. Psychosom. Res..

[29] Chang S.J., Koh S.B., Kang M.G., Hyun S.J., Cha B.S., Park J.K., Park J.H., Kim S.A., Kang D.M., Chang S.S. (2005). Correlates of self-rated fatigue in Korean employees. J. Prev. Med. Public Health.

[30] Sohn S.I., Kim D.H., Lee M.Y., Cho Y.W. (2012). The reliability and validity of the Korean version of the Pittsburgh Sleep Quality Index. Sleep Breath..

[31] Mollayeva T., Thurairajah P., Burton K., Mollayeva S., Shapiro C.M., Colantonio A. (2016). The Pittsburgh Sleep Quality Index as a screening tool for sleep dysfunction in clinical and non-clinical samples: A systematic review and meta-analysis. Sleep Med. Rev..

[32] Yoon D.H., Han G.-Y., Hwang S.S., Lee D.W., Kim J.-S., Kim K., Kim J., Song W. (2020). The effect of fermented porcine placental extract on fatigue-related parameters in healthy adults: A double-blind, randomized, placebo-controlled trial. Nutrients.

[33] Borg G.A. (1982). Psychophysical bases of perceived exertion. Med. Sci. Sports Exerc..

[34] Edvardsen E., Hem E., Anderssen S.A. (2014). End criteria for reaching maximal oxygen uptake must be strict and adjusted to sex and age: A cross-sectional study. PLoS ONE.

[35] Cabral-Santos C., Gerosa-Neto J., Inoue D.S., Panissa V.L.G., Gobbo L.A., Zagatto A.M., Campos E.Z., Lira F.S. (2015). Similar anti-inflammatory acute responses from moderate-intensity continuous and high-intensity intermittent exercise. J. Sports Sci. Med..

[36] Holm S. (1979). A simple sequentially rejective multiple test procedure. Scand. J. Stat..

[37] Schaefer A., Piquard F., Geny B., Doutreleau S., Lampert E., Mettauer B., Lonsdorfer J. (2002). L-arginine reduces exercise-induced increase in plasma lactate and ammonia. Int. J. Sports Med..

[38] Gambardella J., Fiordelisi A., Spigno L., Boldrini L., Lungonelli G., Di Vaia E., Santulli G., Sorriento D., Cerasuolo F.A., Trimarco V. (2021). Effects of chronic supplementation of L-arginine on physical fitness in water polo players. Oxidative Med. Cell. Longev..

[39] Robergs R.A., Ghiasvand F., Parker D. (2004). Biochemistry of exercise-induced metabolic acidosis. Am. J. Physiol.-Regul. Integr. Comp. Physiol..

[40] Alba-Roth J., Müller O.A., Schopohl J., von Werder K. (1988). Arginine stimulates growth hormone secretion by suppressing endogenous somatostatin secretion. J. Clin. Endocrinol. Metab..

[41] Obál F., Krueger J.M. (2004). GHRH and sleep. Sleep Med. Rev..

[42] Elango R. (2023). Tolerable upper intake level for individual amino acids in humans: A narrative review of recent clinical studies. Adv. Nutr..

[43] Esbjörnsson-Liljedahl M., Sundberg C.J., Norman B., Jansson E. (1999). Metabolic response in type I and type II muscle fibers during a 30-s cycle sprint in men and women. J. Appl. Physiol..

[44] Freund H., Oyono-Enguéllé S., Heitz A., Marbach J., Ott C., Zouloumian P., Lampert E. (1986). Work rate-dependent lactate kinetics after exercise in humans. J. Appl. Physiol..

[45] Juel C. (1997). Lactate-proton cotransport in skeletal muscle. Physiol. Rev..

[46] Messonnier L.A., Emhoff C.-A.W., Fattor J.A., Horning M.A., Carlson T.J., Brooks G.A. (2013). Lactate kinetics at the lactate threshold in trained and untrained men. J. Appl. Physiol..

